# A mechano-gated ionic diode enables low-power synaptic tactile spiking

**DOI:** 10.1126/sciadv.aea5656

**Published:** 2025-12-10

**Authors:** Yong Min Kim, Hanbin Choi, Ji Hong Kim, Jin Han Kwon, Elvis K. Boahen, Neul Kyum Ha, Zhengyang Kong, Hong Chul Moon, Do Hwan Kim

**Affiliations:** ^1^ Department of Chemistry, Stanford University, Stanford, CA 94305, USA.; ^2^ Department of Chemical Engineering, Hanyang University, Seoul 04763, Republic of Korea.; ^3^ Department of Chemical and Biomolecular Engineering, Korea Advanced Institute of Science and Technology (KAIST), Daejeon 34141, Republic of Korea.; ^4^ Institute of Nano Science and Technology, Hanyang University, Seoul 04763, Republic of Korea.; ^5^ Clean-Energy Research Institute, Hanyang University, Seoul 04763, Republic of Korea.

## Abstract

Ionic diodes, which enable unidirectional ion transport, hold great promise for adaptive and energy-efficient ionotronic systems. However, conventional designs often rely on high ion concentrations, which promote ion pairing and clustering, thereby reducing mobility and impeding the formation of stable ion depletion layers (IDLs). Here, we report a mechano-gated ionic diode with balanced ionic conductivity between cationic and anionic polymer layers, achieved through copolymer engineering. This conductivity matching enables the formation of well-defined IDLs, yielding a record-high rectification ratio of 23.5 and pressure-sensitive piezo-ionic behavior. The device transduces mechanical stimuli into discrete ionic spikes via threshold-gated current modulation, consuming only 0.41 nanojoules per spike at rest and 1.49 nanojoules under pressure, achieving up to a 24-fold enhancement in signal-to-noise ratio. When integrated into a tactile interface, the diode exhibits synaptic-like plasticity and activity-dependent signal encoding. These findings establish a material-driven strategy for real-time, low-power ionic sensing and neuromorphic functionality.

## Teaser

A mechano-gated ionic diode with balanced conductivity enables low-power synaptic tactile spiking and neuromorphic sensing.

## INTRODUCTION

Ionotronic devices, which exploit ions as charge carriers, have emerged as promising platforms for next-generation device platforms due to their mechanical deformability, low-voltage operation, and seamless integration with biological systems (*1*–*8*). Their capacity to emulate biological signal transduction makes them particularly appealing for adaptive bioelectronic systems, including neuromorphic computing and tactile sensing. However, conventional ionic tactile sensors, such as resistive and capacitive types, rely on passive mechanisms, such as geometric deformation or dielectric modulation, which restrict their responsiveness to dynamic stimuli and require continuous power input (*9*, *10*). To overcome these limitations, organic electrochemical transistors (OECTs) have been suggested, leveraging active ion-electron coupling and signal amplification. Although OECTs enable dynamic signal processing, their use in real-time synaptic systems is limited by slow ion transport, structural instability arising from ion infiltration, and poor control over ion flow (*11*, *12*).

Ionic diodes have recently emerged as promising alternatives, enabling spontaneous and directional ion transport via interfacial charge redistribution without the need for external gating. Constructed from cationic polymer (CP) and anionic polymer (AP) layers, these devices form ion depletion layer (IDL) at their interface, serving as ionic version of semiconductor p-n junctions (*13*, *14*). The resulting unidirectional ion flow renders ionic diodes particularly attractive for pressure-responsive and neuromorphic applications, where selective activation and polarity-dependent signaling are crucial. Although recent studies have demonstrated their responsiveness to mechanical and chemical stimuli (*15*, *16*), overall performance is limited by unstable IDLs, low rectification ratios, and insufficient mechanical adaptability, all originating from the lack of systematic material design and interface engineering strategies (*17*–*20*).

**Fig. 1.**
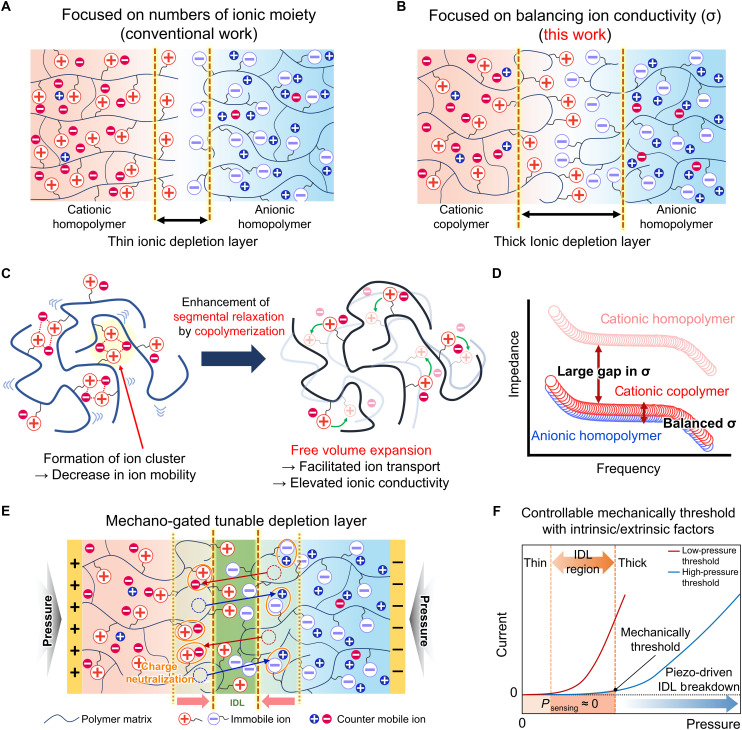
Concepts of material design and characterization for tunable ionic depletion layer of ionic diode. Schematic comparison of IDL formation strategy between (**A**) conventional approaches and (**B**) the present study. Conventional works emphasize increasing the number of ionic moieties, inadvertently reducing ion mobility and thus limiting IDL thickness. In contrast, this study strategically balances ionic conductivity between CP and AP, optimizing IDL thickness and enhancing overall device performance. (**C**) Schematics of chain relaxation motion in homopolymer and copolymer of CP and (**D**) conceptual Bode plot, illustrating this study’s goal: While neat CP exhibits a large conductivity gap compared to AP, cationic copolymers achieve a balanced conductivity comparable to AP. (**E**) Schematic illustration of the tunable IDL region and promoted ionic breakdown phenomenon induced by piezo-ionic behavior. (**F**) Tactile perception performance with controllable mechanically thresholds, regulated by intrinsic (material properties) and extrinsic factors (applied voltage).

The formation of a stable IDL critically depends on rapid and reversible ion redistribution at the CP-AP interface, which is strongly influenced by the ionic conductivity (σ) of each polymer layer. According to the Nernst-Einstein equation ($\sigma =\sum_{i}\mu_{i}z_{i}n_{i}e$, where $z_{i}$ ionic charge and *e* is the elementary charge), σ is determined by both ion mobility (μ_i_) and ion concentration (*n*_i_). To improve these parameters, previous studies have used strategies such as using polyelectrolyte hydrogels or increasing ion content (*14*, *15*, *18*, *20*–*22*). Hydrogels enhance ion diffusivity through water-mediated ion solvation and electrostatic screening, but their practical use is limited by evaporation and poor electrochemical stability. Conversely, increasing the ion content increases the mobile charge carrier density, but it inevitably strengthens ion-polymer and ion-ion interactions, promoting ion pairing and clustering and thereby reducing the effective number of free carriers (*23*, *24*). This paradoxical reduction in free mobile ions ultimately undermines the formation of a robust IDL (Fig. 1A). Notably, many prior studies have focused on maximizing ion content without fully accounting for such effects on ionic dissociation and interfacial redistribution. To overcome these issues, solvent-free polyelectrolytes incorporating bulky ionic groups (e.g., imidazolium or trifluoromethanesulfonimide) have been developed to weaken electrostatic interactions (enhancing ion concentration) and disrupt polymer chain packing (improving ion mobility), thereby increasing ionic conductivity (*25*, *26*).

Despite recent advances in tuning conductivity within individual polymers, conductivity imbalance between the CP and AP components of ionic diodes remains largely unresolved. This disparity originates from the intrinsic structural asymmetry of the counterions tethered to each polymer: APs are typically paired with small, mobile cations, whereas CPs are coupled with bulky, less mobile anions, resulting in a fundamental difference in conductivity. Ionic conductivity is dictated by both the concentration of free carriers and their intrinsic mobility, which, in turn, depend on counterion dissociation and polymer chain dynamics. In other words, high conductivity reflects efficient dissociation of coion/counterion pairs, producing a large population of free ions. This dissociation not only facilitates bulk transport but also ensures that free coions are exposed at the interface, a prerequisite for stable IDL formation. When the conductivities of CP and AP are mismatched, the redistribution of counterions across the junction becomes asymmetric, destabilizing the interfacial electrochemical equilibrium and hindering robust IDL establishment. We therefore hypothesize that balancing conductivity between oppositely charged polymers is essential for efficient IDL formation and enhanced rectification performance. This concept parallels design strategies in organic electronics, where balanced charge-carrier mobilities are critical for effective transport and recombination (*27*, *28*).

Here, we report a dynamic ionic diode platform with balanced conductivity that transduces mechanical stimuli into discrete, directionally modulated ionic signals for tactile and neuromorphic applications. To this end, we proposed copolymer-based engineering to systematically tune the conductivity and chain dynamics of the CPs (Fig. 1B). Copolymerizing low-glass transition temperature (*T*_g_) monomers (e.g., butyl acrylate) enhanced overall segmental mobility and increased free volume, which suppressed ion clustering conducive to improving ion mobility, thereby resulting in elevated overall ionic conductivity to match that of the AP (Fig. 1C). This conductivity matching enables the formation of a thick, uniform IDL across the CP-AP bilayer, resulting in markedly improved rectification performance (Fig. 1D). Notably, the optimized bilayer exhibits mechano-gated behavior, generating distinct output signals only when applied pressure exceeds a defined threshold (Fig. 1E). This threshold-gated response enables continuous “on” states with minimal energy consumption, in contrast to devices without a well-defined IDL formation, which show linear responses requiring constant power input (Fig. 1F). The resulting pressure-dependent signal modulation supports ionic processing, in which only meaningful mechanical inputs trigger discrete spikelike signals. This threshold-gated behavior eliminates unnecessary energy expenditure and, notably, provides superior energy efficiency compared to conventional memristor- and transistor-based signal processing devices under pressure stimuli. Thus, the optimized diode not only enhances rectification but also enables threshold-triggered current modulation, key features for tactile sensing and synaptic plasticity.

## RESULTS

### Control of ionic transport and rectification via polymer chain dynamics

To investigate how ionic conductivity and polymer structure influence IDL formation and rectification, we began by tuning the conductivity of each ionic layer in the bilayer system. We first modulated AP by exchanging its counterion with a bulky, low-diffusivity cation, butyl methylimidazolium ([BMIM^+^]), which substantially decreased its conductivity (fig. S1). This tuning narrowed the ionic conductivity gap between the AP and the CP. However, the resulting bilayer exhibited poor IDL formation and a notable reduced rectification ratio of 2.1, indicating that conductivity matching alone is insufficient without maintaining high absolute ionic transport. Next, we examined the influence of counterion pairing in the CP by substituting various anions. Because smaller anions are generally expected to enhance diffusivity and conductivity, we introduced hexafluorophosphate ([PF_6_^−^]) into the CP to probe its effect. Contrary to this expectation, PF_6_^−^ exhibited strong electrostatic association with the quaternary ammonium sites, which increased ion pairing and thereby reduced both ionic conductivity and rectification (fig. S2). In contrast, bulkier and charge-delocalized anions such as (bis(trifluoromethanesulfonyl)imide) ([TFSI^−^]) weakened these interactions, suppressed ion pairing, and enhanced ion transport, leading to improved rectification performance. Despite this improvement in conductivity of CP, a notable mismatch with AP still remained, continuing to hinder efficient IDL formation and rectification.

Therefore, we fixed the counterions to ethyl methylimidazolium ([EMI^+^]) in the AP and [TFSI^−^] in the CP, both providing optimal ionic mobility, and introduced copolymers as a strategy to simultaneously improve ionic conductivity and interfacial compatibility. Specifically, we synthesized a series of cationic copolymers (CP70, CP40, and CP10) containing varying fractions of a flexible, low-*T*_g_ comonomer to tune segmental mobility while maintaining a sufficient density of ionic groups (see note S1, figs. S3 to S7, and table S1). Incorporation of soft segments substantially reduced polymer rigidity and decreased the segmental relaxation time (τ_e_). Notably, CP40 exhibited τ_e_ values comparable to those of the AP, suggesting well-matched chain dynamics across the CP-AP interface (fig. S8). Although the incorporation of neutral comonomers reduced the overall *n*_i_ in CP, the substantial enhancement in chain mobility more than compensated for this decrease, leading to a net increase in conductivity value (fig. S9). However, excessive incorporation of nonionic segments, as in the CP10, led to a sharp decline in conductivity, emphasizing the need to balance polymer flexibility and ionic charge density for optimal performance.

**Fig. 2.**
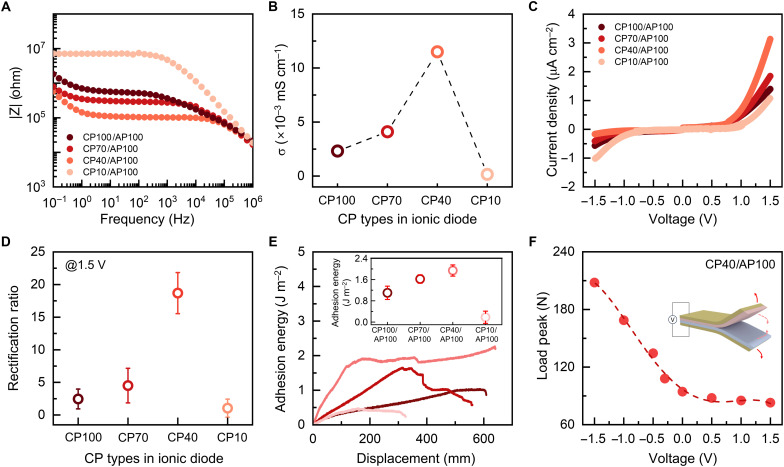
Control of ionic transport and rectification via polymer chain dynamics in ionic diode. (**A**) Bode plot of ionic diode and (**B**) corresponding σ extracted using following equation: σ = h/AR, where h, A, and R represent thickness, contact area between the CP or AP, and bulk resistance, respectively. (**C**) *I*-*V* curves of ionic diode based on different CP type and (**D**) obtained rectification ratio of ionic diode at application of 1.5 V. (**E**) Adhesion energy–displacement curves measured from 180° peel off test. Inset: Adhesion energy value, defined as the plateau peeling force divided by the sample width (2F_plateau_/*W*). (**F**) Maximum load peak as respective of applied voltage obtained from fig. S14.

To investigate how polymer chain dynamics influence diode performance, we fabricated ionic diodes using each CP variant. Because of the inherently higher ionic conductivity of the AP, the impedance of the CP-AP heterojunction closely matched that of the CP homojunction, confirming that ion transport was primarily limited by the CP phase (Fig. 2, A and B). Notably, ionic diode based on CP40 exhibited the most favorable interfacial characteristics, as evidenced by the highest interfacial resistance (R_IDL_) and the largest IDL capacitance (C_IDL_), indicating enhanced charge storage and the formation of a well-defined IDL (fig. S10, table S2, and note S2). Under applied bias, these devices also showed pronounced phase angle modulation, reflecting dynamic reconfiguration of the IDL driven by directional ion migration (fig. S11). The reduction in the low-frequency phase angle under forward bias further supported enhanced interfacial ion mobility. Moreover, the CP40-based bilayer maintained effective ionic rectification and electrochemical stability up to 1.5 V, without initiating Faradaic reactions, demonstrating voltage-tolerant operation of the solvent-free ionic elastomer matrix (Fig. 2C). Consequently, the CP40-based ionic diode achieved a rectification ratio of 23.5 ± 2.2, the highest reported for solvent-free polyelectrolyt–based IDL diodes (Fig. 2D). In contrast, double-layered CP40 or AP100 homojunction devices showed negligible rectification, highlighting the critical role of interfacial design and ionic conductivity balancing in enabling high rectification efficiency (fig. S12). To confirm that these rectification behaviors indeed originate from IDL formation, we performed attenuated total reflectance–Fourier transform infrared (ATR-FTIR) spectroscopy at each electrode interface. Characteristic vibrational signatures of TFSI^−^ were detected on both sides of the junction, demonstrating that ions from the AP and CP layers are predistributed across the interface (fig. S13). Notably, clear TFSI^−^ peaks were observed on the AP side, although such signals are absent when the AP is measured alone. This provides direct spectroscopic evidence that counterions penetrate across the interface, corroborating the formation of a well-defined IDL that underpins the observed diode behavior.

Because the IDL is closely tied to physical interactions, we further conducted contact adhesion tests to evaluate the interfacial adhesiveness of the ionic diode (Fig. 2E). Results of 180° peel test indicated that adhesion energy increased with stronger interchain interactions and polymer intermingling within IDL. The CP40-based ionic diode showed the highest adhesion energy (1.94 ± 0.21 J·m^−2^), attributed to its balanced chain mobility and interfacial compatibility. In contrast, the CP10-based ionic diode exhibited the lowest adhesion energy (0.18 ± 0.23 J·m^−2^), suggesting that insufficient interchain interactions hinder IDL expansion. Moreover, the voltage-dependent increase in adhesion energy under reverse bias in the CP40-based ionic diode indicates that IDL expansion strengthens interfacial electrostatic interactions (Fig. 2F and fig. S14).

### Mechanically controlled ionic transport and rectification behavior

**Fig. 3.**
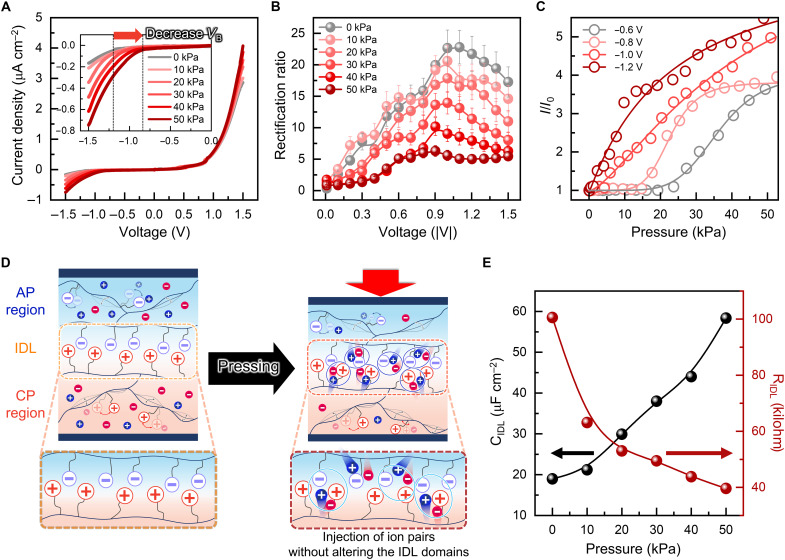
Mechanically controlled ionic transport and rectification behavior in ionic diode. (**A**) *I*-*V* characteristics of CP40-based ionic diode under varying pressures, showing increased current density, especially in reverse bias. Inset: *V*_B_ decreases with higher pressure. (**B**) Rectification ratio changes under varying pressure, and (**C**) current modulation under reverse bias. (**D**) Schematics of the piezo-ionic mechanism: Pressure-driven ion migration modifies rectification without changing IDL thickness. (**E**) Pressure-dependent changes in C_IDL_ and R_IDL_.

To move beyond conventional voltage-driven control, we explored mechanical tuning of the IDL as a strategy for dynamically modulating ionic diode behavior. Specifically, we investigated whether pressure-induced ionic redistribution could restructure the IDL and trigger rectification switching in response to mechanical input. As a conceptual framework, we introduced a piezo-ionic mechanism, wherein externally applied pressure alters ion migration and accumulation at the CP-AP interface. To validate this concept, we applied incremental pressure to the CP40-based ionic diode. The applied mechanical stress promoted ion migration toward the junction, enriching the IDL with mobile ions and thereby increasing the current across the device (Fig. 3A). At the same time, the ionic breakdown voltage (*V*_B_) was markedly reduced, from −1.18 V at 0 kPa to −0.80 V at 50 kPa. This response is consistent with the piezo-ionic principle, in which mechanical force reshapes the electrochemical potential landscape and drives ion redistribution (*29*, *30*). Furthermore, the rectification ratio also decreased notably, dropping from 21.73 ± 2.79 under no applied pressure to 5.01 ± 0.72 at 50 kPa. This demonstrates that the diode’s switching characteristics become increasingly sensitive to mechanical force (Fig. 3B).

The modulation of electrical characteristics by pressure highlights the potential for dynamic, input-dependent control of conduction states, an essential feature for tactile sensing. Under combined pressure and voltage stimuli, the device exhibited high sensitivity and threshold behavior, generating current only when both inputs exceeded critical levels (Fig. 3C and fig. S15), where this threshold-dependent current generation leads to a distinct change in pressure sensitivity across the threshold. In contrast, this behavior was absent under forward bias (fig. S16) or in homojunction systems lacking a well-developed IDL (fig. S17). Moreover, ionic diode based on CP100, which forms a narrower IDL, showed a negligible pressure response and sensitivity variation, further supporting the critical role of initial IDL structure in enabling mechanically gated ion transport (fig. S18).

To elucidate the mechanism underlying piezo-ionic modulation, we systematically examined how electrical bias and mechanical pressure influence the IDL properties, focusing on two derived metrics: C_IDL_ and R_IDL_ (fig. S19 and table S3). Under forward bias, mobile counterions migrate into the IDL and neutralize resident coions, which weakens local electrostatic interactions and reduces charge asymmetry at the interface. This leads to a decrease in both C_IDL_ and R_IDL_ (fig. S20), indicating diminished IDL. In contrast, reverse bias extracts mobile ions from the junction region, enhancing charge imbalance and thereby thickening the IDL, as reflected by increased R_IDL_, a hallmark of rectification in ionic diodes (*14*). Unlike voltage-driven migration, which drives unidirectional ion movement based on polarity, applied pressure induces the simultaneous infiltration of neutral ion pairs into the IDL. This pressure-driven redistribution increases local ion concentration while maintaining electrostatic neutrality, thereby modulating the IDL structure without altering net charge (Fig. 3D). Experimental results corroborated this interpretation, as pressure application led to a pronounced increase in C_IDL_ and a concomitant decrease in R_IDL_ (Fig. 3E, fig. S21, and table S4), indicative of ion-pair accumulation and pressure-induced structural reorganization within the IDL. Furthermore, short- and open-circuit tests revealed that pressure-induced current was negligible compared to voltage-driven conduction (fig. S22), confirming that pressure modulates the internal ionic landscape without acting as a primary electrical driving force.

### Dynamic synaptic plasticity in ionic diode: A pathway to tactile neural devices

To highlight the scalability of our approach, we first note that ionic diodes can be readily fabricated through monomer-solution photopolymerization, which allows on-demand patterning in arbitrary sizes and geometries. Devices with lateral dimensions of 1 cm by 1 cm and 3 cm by 3 cm exhibited nearly identical *I*-*V* characteristics and rectification ratios (fig. S23), confirming size-independent operation and seamless scalability. Equally important, the ionic polymer matrix demonstrates excellent intrinsic stability. Thermogravimetric analysis (TGA) revealed negligible weight loss (<1%) near 100°C for both AP and CP membranes (fig. S24), indicating the absence of measurable water absorption and robust thermal integrity.

**Fig. 4.**
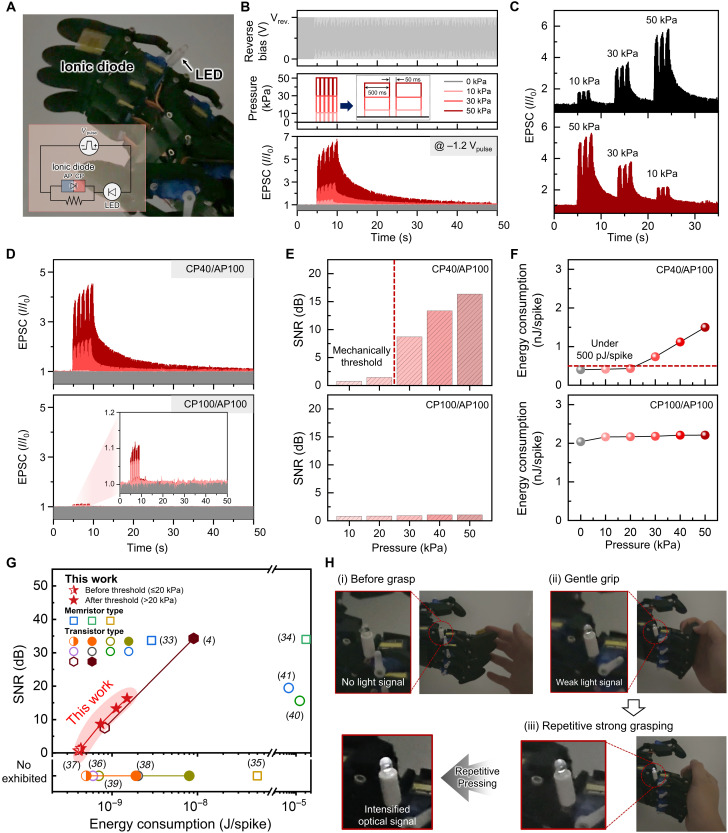
Dynamic synaptic plasticity in ionic diode: A pathway to tactile neural devices. (**A**) Photograph of the fully assembled artificial tactile system, featuring a robotic hand with a ionic diode connected to an LED bulb. (**B**) Pressure-sensitive current modulation under reverse bias pulses. (**C**) Stepwise pressure input demonstrating synaptic plasticity. Comparison of (**D**) tactile sensing, (**E**) signal-to-noise ratio (SNR), and (**F**) energy consumption between CP40- and CP100-based ionic diodes under repeated pressure. (**G**) Comparison plot of synaptic energy consumption with SNR in current tactile-sensory devices: open symbols, no pressure; half filled, low pressure; filled, high pressure. (**H**) Demonstration of pressure-sensitive response: Weak pressure (gentle grasp) yields negligible signals; stronger pressure increases signal intensity, progressively brightening the LED.

Building on this principle, we fabricated ionic diodes by sandwiching CP40 and AP100 layers between flexible indium tin oxide (ITO)–polyethylene terephthalate (PET) films and conformally attaching them onto robotic fingers. We then integrated these devices into a robotic hand equipped with an light-emitting diode (LED) indicator (Fig. 4A), thereby constructing a tactile interface that enabled both the application and visualization of mechanical inputs. Under pulsed reverse bias, pressure stimuli induced distinct current modulation confined to individual tactile inputs (Fig. 4B), allowing real-time tracking of synaptic modulation. Unlike prior ionic diodes, which were largely limited to electrical synaptic behaviors such as paired-pulse facilitation and faced challenges in tactile integration (*13*), our device exhibited activity-dependent synaptic modulation triggered by pressure. Unlike general ionic diodes that produce repetitive outputs without residual modulation (*31*, *32*), our device exhibited dynamic current adaptation, a feature essential for encoding variable mechanical inputs. The same threshold-tunable behavior observed electrically (Fig. 3C) was also reproduced under mechanical stimuli, as shown in movie S1, underscoring the ability of ionic diodes to convert pressure into neuromorphic outputs.

The applied pressure increased the residual ion concentration within the IDL, thereby facilitating current modulation induced by ionic breakdown and further amplifying synaptic plasticity under pulsed reverse bias (fig. S25). This enhancement in synaptic plasticity, driven by ionic current modulation, originates from pressure-induced IDL reconfiguration and dynamic ion transport under repeated mechanical and electrical inputs. This effect was absent under constant dc bias or in homojunction configurations (figs. S26 and S27). Under constant dc bias, the electrostatic field restricts further ion redistribution or accumulation within the IDL, resulting in uniform ionic breakdown currents. Furthermore, in the absence of an IDL region, the device operates akin to a conventional ion gel system that relies solely on ion diffusion to form electrical double layer. These observations underscore the critical role of a well-defined, charge-separated IDL in enabling piezo-ionic synaptic modulation. We further evaluated its response to different tactile input parameters, including the number of applied pressure pulses, interval time, and duration. With increasing pulse numbers, the degree of plasticity and current modulation increased substantially compared to equivalent continuous pressure (fig. S28). The added benefit of this piezo-ionic mechanism lies in its capacity to progressively amplify signals through the accumulation of residual ions in the IDL following repeated or stronger pulses, while single or weak inputs are effectively filtered out. Moreover, the relationship between current modulation ratio, pulse interval, and duration under varying pressure conditions exhibited a trend analogous to that of neural devices responding to electrical pulses (fig. S29). Thus, the ionic diode demonstrated reliable synaptic plasticity even under dynamically varying stepwise pressure inputs (Fig. 4C), reinforcing its practical potential in tactile neural interfaces.

To gain depper insight into the role of a thick IDL in tactile-dependent synaptic plasticity, we compared CP40- and CP100-based ionic diodes under identical conditions. Devices incorporating CP40, which exhibited conductivity balance between AP and CP layer, thereby, optimized IDL thickness, showed markedly stronger pressure-induced synaptic responses than that of CP100 system (Fig. 4D, fig. S30, and movie S2). These synaptic plasticity responses were preserved regardless of device size, which we attribute to the presence of a well-defined IDL that governs ion redistribution (fig. S31). This intrinsic mechanism ensures uniform behavior across different footprints, thereby providing a solid basis for scalability. This feature resulted in a substantial improvement in signal-to-noise ratio (SNR): Whereas CP100-based devices exhibited negligible improvement (~0.4 dB), CP40-based systems (16.32 dB) showed more than a 24-fold increase (Fig. 4E and fig. S32).

In addition, CP40-based ionic diode consumed substantially less energy under low-pressure conditions, making them ideal for applications requiring long idle periods with minimal power draw (Fig. 4F and fig. S33). When benchmarked against recently reported memristor- and transistor-based tactile-synaptic systems (Fig. 4G and table S5), our system exhibited the lowest standby energy consumption (0.41 nJ/spike) before pressure activation. Unlike previous systems (*4*, *33*–*41*) that show nearly 10-fold consumption energy increases upon stimulation, our devices maintained high SNR with only 1.49 nJ/spike, demonstrating pressure-sensitive operation without compromising efficiency. This dual functionality, low standby power and pressure-tunable responsiveness, marks a notable step toward practical neuromorphic tactile platforms. To showcase this capability, we constructed a live demonstration circuit (Fig. 4H and movie S3). The device remained inactive under weak touch, conserving energy, but generated progressively brighter LED signals with increasing pressure and repetition, validating its use as a responsive, energy-efficient synaptic interface.

## DISCUSSION

This work presents a dynamic ionic diode that exhibits high rectification and pressure-triggered current modulation, enabling threshold-dependent ionic response to mechanical stimuli. To realize this functionality, we used a copolymer-engineering strategy that enhanced polymer segmental mobility and balanced ionic conductivity between oppositely charged polymers, thereby promoting the formation of a robust, tunable IDL and achieving a high rectification ratio of 23.5. In contrast to conventional passive ionic tactile sensors, and surpassing advanced OECT-based systems, the diode demonstrates low energy consumption (0.41 nJ/spike at rest and 1.49 nJ/spike under pressure) and a high SNR (16.3 dB) during synaptic-like signal encoding. This piezo-ionic behavior enables mechanical stimuli to be transduced into discrete, adaptive ionic outputs with minimal standby power, addressing critical limitations in neuromorphic tactile systems. When integrated into a tactile interface, the device exhibits activity-dependent plasticity and real-time pressure perception, validating its potential for intelligent sensory platforms. By linking molecular material design to macroscale responsiveness, this study provides a generalizable strategy for low-power, pressure-sensitive bioelectronics and next-generation soft robotics.

## MATERIALS AND METHODS

### Materials

3-(acryloylaminopropyl)trimethylammonium chloride solution (448281), butyl acrylate (234923), 2-acrylamido-2-methyl-1-propanesulfonate (655821), 1-ethyl-3-methylimidazolium bromide (89483), lithium bis(trifluorosulfonyl)imide (919977), *N*,*N*′-methylenebisacrylamide (146072), 2-hydroxy-4′-(2-hydroxyethoxy)-2-methylpropiophenone (410896), 2-hydroxy-2-methylpropiophenone (405655), poly(ethylene glycol) diacrylate (455008), 1-butyl-3-methylimidazolium chloride (94128), and acetonitrile (34851) were purchased from Sigma-Aldrich. Deionized water (high-performance liquid chromatography) was purchased from J.T.Baker (New Jersey, USA). ITO-coated PET substrate (sheet resistance: 60 ohm square^−1^) was purchased from Sigma-Aldrich.

### Fabrication of CP-AP complexed ionic device

The ionic diode, designed with a sandwich structure to establish a bilayer junction interface, was fabricated through a two-step process. First, a film of cationic copolymer (or anionic homopolymer) was gently laminated onto an ITO-coated PET substrate. The laminated film underwent thermal treatment at 60°C for 1 hour to ensure uniform adhesion. Subsequently, the bilayer junction of CP and AP was prepared by simply attaching the two bare surfaces of the laminated films. This bilayer junction was then subjected to an additional thermal annealing step at 60°C for 1 hour, completing the fabrication of ionic diode. All thermal treatment steps following lamination were conducted above the glass transition temperature (*T*_g_) of the respective ionic polymers to ensure optimal interfacial adhesion. The fabrication process was consistently applied to ionic diodes, incorporating a series of cationic copolymers synthesized with varying compositions, including cationic homopolymer, to systematically investigate the influence of CP composition on the ionic diode characteristics. Furthermore, homojunction devices composed solely of cationic copolymers or anionic homopolymers were manufactured following the same fabrication process. Unless otherwise specified, all devices were fabricated with a consistent area of 10 mm by 10 mm for uniform evaluation. Detail procedures for preparation of monomers are described in note S1.

### Characterizations

The ^1^H, ^19^F nuclear magnetic resonance spectra were recorded on an Avance III HD 500 instrument using D_2_O or CDCl3 (99.9%; Sigma-Aldrich) as a solvent. The number of average molecular weight (*M_n_*) and polydispersity (*Đ*) of the used polymer was characterized by size exclusion chromatography (LC-4500, JASCO) with a refractive index detector (RI-4030, JASCO) calibrated with standard poly(ethylene oxide) (calibration kit, Scientific Polymer Products Inc.). The FTIR spectroscopy was evaluated using the ATR technique (Spectrum 100, PerkinElmer). The *T*_g_ values of the prepared ionoconductors were measured by differential scanning calorimetry (DSC; DSC 4000, PerkinElmer) under N_2_ atmosphere. The sample (typical weight ~ 6.0 mg) was first heated to 150°C and held at this temperature for 20 min to eliminate its thermal history, followed by quenching at −30°C. The DSC thermograms were recorded during a second heating stage at a rate of 10°C min^−1^. Thermal stability analysis was conducted using a thermal analyzer (SDT 650, TA Instruments). The temperature was increased from 25° to 600°C at a rate of 10°C min^−1^ under N_2_ atmosphere. The viscoelastic characterizations were characterized by an oscillatory rheometer (MCR-92, Anton Paar) with 8-mm parallel plates. The sample gap was fixed at ~1 mm, and the strain was set to a small strain amplitude (1%) for frequency-dependent measurements. TGA 7 (PerkinElmer) was performed on samples equilibrated in air at 50% relative humidity for 24 hours. Approximately 5 mg of each sample was placed in a platinum pan and heated from room temperature to 500°C at a rate of 10°C min^−1^.

Adhesive strength measurements were conducted using a vertically motorized test stand (ESM303, Mark-10) equipped with a force gauge (M5-10, Mark-10). CP and AP samples were prepared via direct photopolymerization onto a supporting PET membrane in a rectangular shape (20 × 10 × 0.2 mm) and cured at 80°C under reduced pressure overnight. The resulting films were then sandwiched together and subjected to thermal treatment in an oven at 50°C for 5 min. Before testing, the specimens were cooled to room temperature and allowed to cure for an additional 24 hours. Adhesion strength was determined as the maximum force (in newtons) sustained by the adhesive joint, normalized by the overlap area (in square millimeters).

Electrochemical impedance spectroscopy (EIS) measurements were performed using an electrochemical analyzer (IM6, ZAHNER) over a frequency range of 10^−1^ to 10^−6^ Hz, applying an ac signal with an amplitude of 10 mV. The ionic conductivity (σ) was calculated using the following equation: σ = h/AR, where h, A, and R represent thickness, contact area between the CP or AP, and bulk resistance, respectively. For electrochemical analysis under forward and reverse bias conditions, the positive terminal (working electrode) was connected to the AP homopolymer, while the negative terminal (counter and reference electrodes) was connected to the CP copolymer. Impedance spectra were also recorded under controlled pressure conditions using a force gauge (M5-2, Mark-10). Experimental EIS data were fitted using ZMan software (ZAHNER, version 2.5). Details of the fits are described in the note S2.

To evaluate the rectifying performance under various bias and pressure conditions, the current-voltage (*I*-*V*) characteristics of the ionic diode were measured using a source meter (Keithley, 2420). The electrical potentiation properties of ionic diodes were characterized using a semiconductor parameter analyzer (Keithley, 4200-SMU). All electrical measurements were conducted on a custom-built sensor probe station (MS-TECH) equipped with a programmable *z*-axis stage and a force gauge (M5-2, Mark-10).

## Supplementary Materials

### The PDF file includes:

Supplementary Notes S1 and S2 Figs. S1 to S33 Tables S1 to S5 Legends for movies S1 to S3 References
