# Supplementary material for: A mechano-gated ionic diode enables low-power synaptic tactile spiking
Source: Sci Adv. 2025 Dec 10;11(50):eaea5656. doi: 10.1126/sciadv.aea5656 (PMC12694019; doi:10.1126/sciadv.aea5656)
Supplement: Supplementary file 1 — Supplementary Notes S1 and S2 Figs. S1 to S33 Tables S1 to S5 Legends for movies S1 to S3 References [file sciadv.aea5656_sm.pdf]

Science Advances
AAAS

Supplementary Materials for

A mechano-gated ionic diode enables low-power synaptic tactile spiking

Yong Min Kim *et al.*

Corresponding author: Hong Chul Moon, hcmoon@kaist.ac.kr; Do Hwan Kim, dhkim76@hanyang.ac.kr

Sci. Adv. **11**, eaea5656 (2025)
DOI: 10.1126/sciadv.aea5656

The PDF file includes:

Supplementary Notes S1 and S2
Figs. S1 to S33
Tables S1 to S5
Legends for movies S1 to S3
References

Other Supplementary Material for this manuscript includes the following:

Movies S1 to S3

Supplementary Note S1.

Fabrication of starting monomer for anionic polymer (AP) and cationic polymer (CP).

[2-Acrylamido-2-methyl-1-propanesulfonate][1-Ethyl-3-methylimidazolium] ([AMP][EMI]) was prepared via an ion-exchange reaction (see fig. S3a). The detailed procedure was as follows: 2-Acrylamido-2-methyl-1-propanesulfonate (30.88 g, 0.13 mol) and an excess amount of 1-ethyl-3-methylimidazolium bromide (45.72 g, 0.24 mol) were each dissolved in acetonitrile (15 g). The two solutions were combined in a round-bottom flask and stirred at room temperature for 12 h under N_2 gas. The byproduct was filtered, and the solvent was removed by evaporation. The AP monomer was obtained as viscous liquid after dried at 40 ºC under reduced pressure. The [2-Acrylamido-2-methyl-1-propanesulfonate] [1-Buthyl-3-methylimidazolium] ([AMP][BMI]) was synthesized following same procedure. The (3-acrylamidopropyl trimethyl ammonium bis(trifluorosulfonyl)imide) ([AA][TFSI]) was prepared via an ion-exchange reaction. The detailed procedure was as follows: a 3-acrylamidopropyl trimethyl ammonium chloride solution (75 wt% in deionized (DI) water) (50 g, 0.18 mol) and an excess amount of lithium bis(trifluorosulfonyl)imide (62.50 g, 0.21 mol) were mixed in DI water (20 g) and stirred at room temperature for 12 h under N_2 gas. The product was extracted from the product solution using dichloromethane, and the extraction was repeated three times. The resulting solution was concentrated by evaporation, and the final viscous liquid (AATFSI) was dried at 40 °C under reduced pressure and sharp TFSI peak was observed at ^{19}F NMR (see fig. S3b).

Preparation of solid-state AP and CPs.

The AP/CP was prepared via in-situ photo-polymerization (fig. S4). A mixture of [AMP][EMI] (10 g, 33 mmol), N,N′-methylenebisacrylamide (crosslinker, 3 mg, 0.019 mmol), and 2-hydroxy-4-(2-hydroxyethoxy)-2-methylpropiophenone (initiator, 0.01 g, 0.04 mmol) was prepared. The prepolymer solution was drop-cast onto a Teflon mold (thickness: 1 mm, width: 10 mm, length: 10 mm). Subsequently, UV irradiation was applied for 60 s at room temperature to form the AP polymer electrolyte. The CPs were synthesized via a route similar to that of the AP polymer electrolyte. The prepolymer solution was prepared by [AA][TFSI] (5.15-1.54 g, 11.4-3.4 mmol), butyl acrylate (monomer, 0-1.204 g, 0-9.4 mmol), poly(ethylene glycol) dimethacrylate (crosslinker, 0.008 g, 0.011 mmol), and 2-hydroxy-2-methylpropiophenone (initiator, 0.015 g, 0.091 mmol). The amount of butyl acrylate was optimized to balance the ionic conductivities of AP and CP by tailoring polymer chain flexibility and ion diffusivity (fig. S5). All CPs were prepared using the aforementioned in-situ photo-polymerization process. The disappearance of the C=C stretching peak which is characteristic of monomer confirms that the polymer was successfully formed and that all monomers reacted completely without any remaining unreacted monomers (fig. S6).

Supplementary Note S2.

EIS-based equivalent circuit model analysis of the junction structures composed of AP and CPs.

To analyze the variations in the ionic depletion layer (IDL) within the heterojunction structure composed of CP and AP under diverse internal (CP composition) and external (electrical and mechanical stimuli) influences, we adopted a previously established equivalent circuit model[13,14]. This model extends a fundamental equivalent circuit previously employed for the analysis of polyelectrolytes[42] by incorporating additional interfacial circuit elements that account for the formation and modulation of IDL at the heterojunction interface. The fundamental equivalent circuit comprises a parallel combination of a bulk resistance (R_B), which represents the transport

of free ions within the bulk region, and a capacitance (C_B) associated with the polarization of ionic monomeric moieties. Additionally, a constant phase element (CPE_{EDL}) is introduced to model the frequency-dependent charge accumulation at the interface, forming the electric double layer (EDL). A contact resistance (R_C) is also included in series with the circuit model to represent interfacial resistance at the electrode-polymer contact.

To accurately represent IDL variations, two additional interfacial circuit elements are incorporated in parallel: CPE_{IDL} representing the IDL capacitance and a resistance (R_{IDL}) accounting for interfacial ionic conduction across the IDL region. These interfacial components are connected in series with the fundamental equivalent circuit to establish a comprehensive model for heterojunction-based ionic diodes. The validity of this equivalent circuit model is further supported by the asymmetric response of phase angle observed at low frequencies in all CP-based ionic diode under applied bias, which correlates with variations in the IDL region (fig. S10)[17,26]. The effective capacitance of the EDL and IDL can be estimated using the Brug model[43,44], based on the parameters extracted from the CPE elements in the equivalent circuit: $C_{EDL} = Q_{EDL}^{1/\alpha_{EDL}} R_B^{(1/\alpha_{EDL}-1)}$, $C_{IDL} = Q_{IDL}^{1/\alpha_{IDL}} R_I^{(1/\alpha_{IDL}-1)}$, where Q is a constant and α is a constant ranging from 0 to 1.

Fitting curves corresponding to various internal and external influences are respectively presented in Figures S10, S19, and S21, while the extracted fitting parameters are summarized in Tables S2, S3, and S4, respectively.

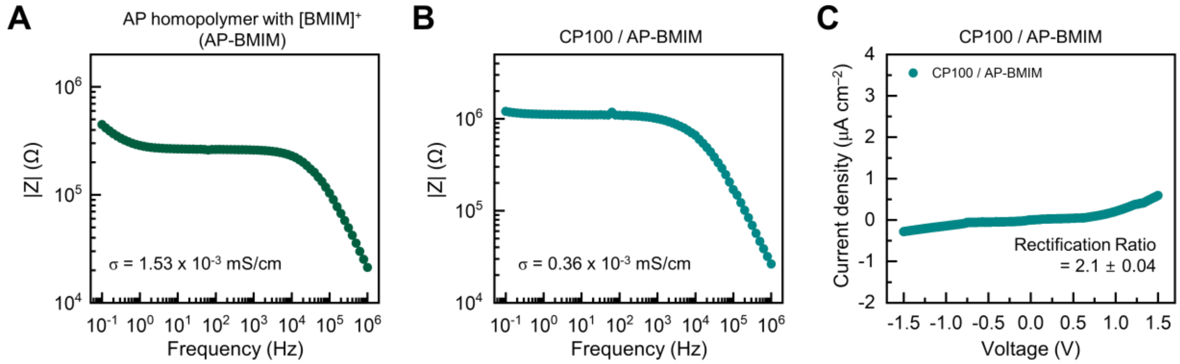

Fig. S1. Changes in conductivity and I-V curve by anion substitution of AP. Bode plot of (**A**) AP-BMIM and (**B**) heterojunction AP-BMIM/CP 100 device. (**C**) I-V curve of AP-BMIM/CP 100 device.

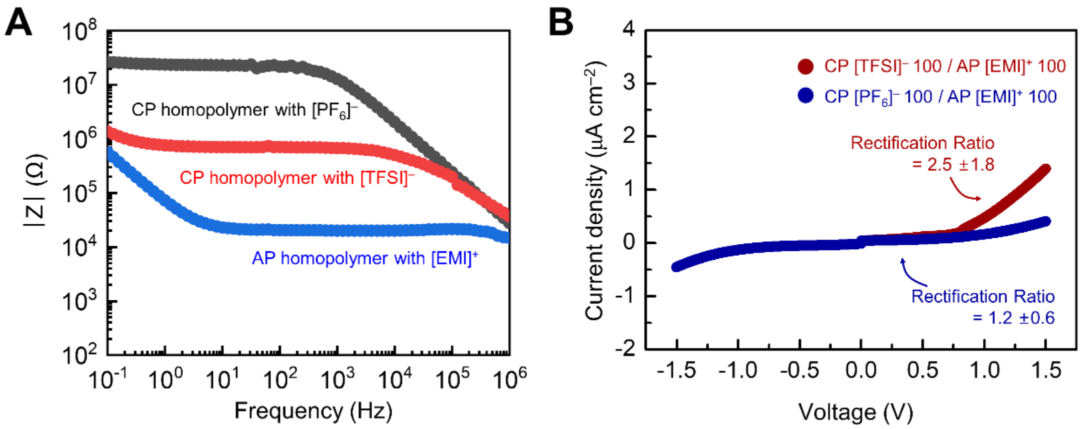

Fig. S2. Influence of counterion n the CP. (A) Ionic conductivity and (B) rectification ratios with varying counterions in CP, showing suppressed rectification for PF_6^- and improved performance with bulkier anions such as TFSI⁻.

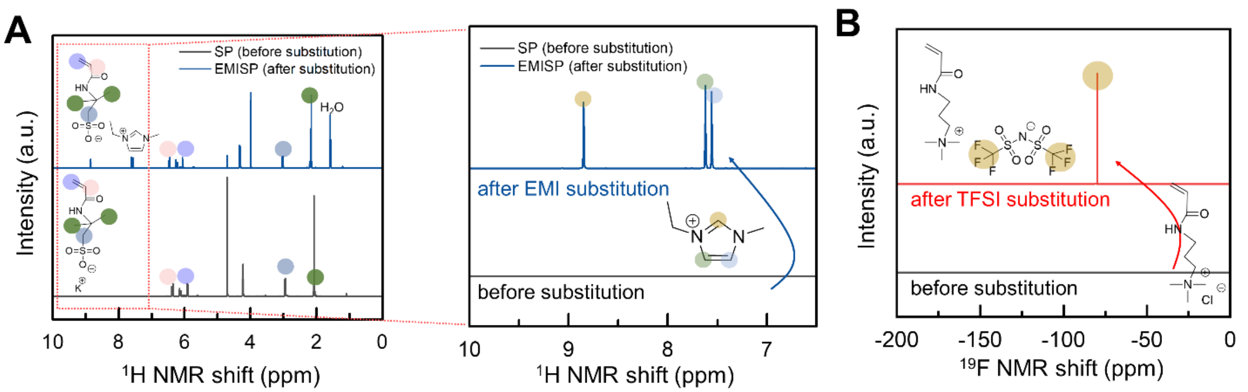

Fig. S3. Confirmation of counter anion substitution of monomers. (**A**) ^{1}H NMR spectra for [AMP][EMI] before and after EMI^{+} exchange and (**B**) ^{19}F NMR for [AA][TFSI] before and after $TFSI^{-}$ substitution.

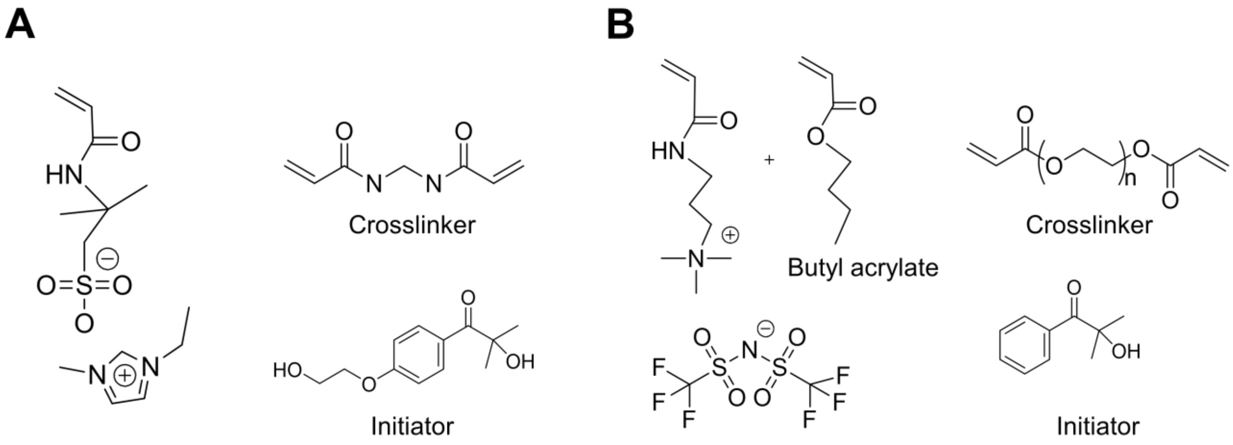

Fig. S4. Molecular structures of components forming the polymers used in this work. (A) AP composed of [AMP][EMI], crosslinker and photoinitiator and (**B**) CPs copolymerized with [AA][TFSI] and butyl acrylate at molar ratios of 0%, 30%, 60%, and 90% for CP 100, CP 70, CP 40, and CP 10, respectively, with a crosslinker and photoinitiator.

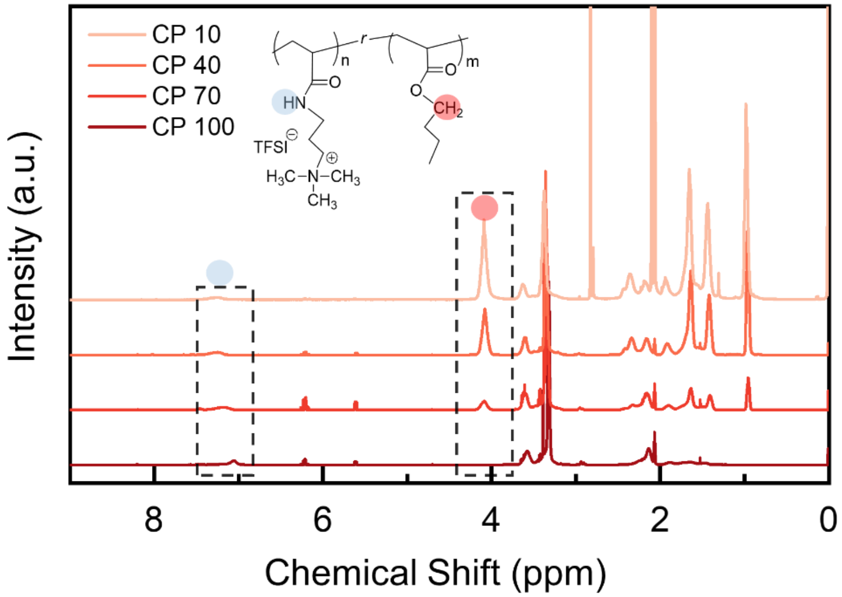

Fig. S5. ^{1}H NMR spectra of synthesized CPs. The molar ratio of each monomer in the copolymers was determined by integrating the characteristic peaks of each monomer.

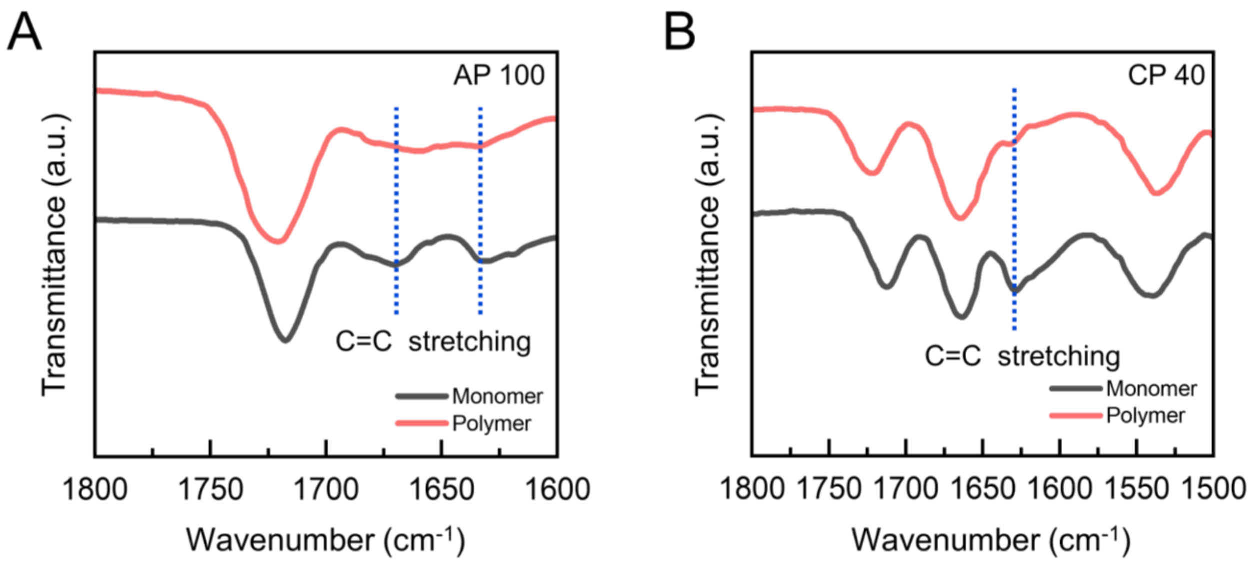

Fig. S6. Confirmation of photo-polymerization. FT-IR spectra of synthesized (**A**) AP 100 and (**B**) CP 40. The disappearance of the characteristic C=C stretching peak of the monomer confirms a high conversion rate, indicating successful polymerization.

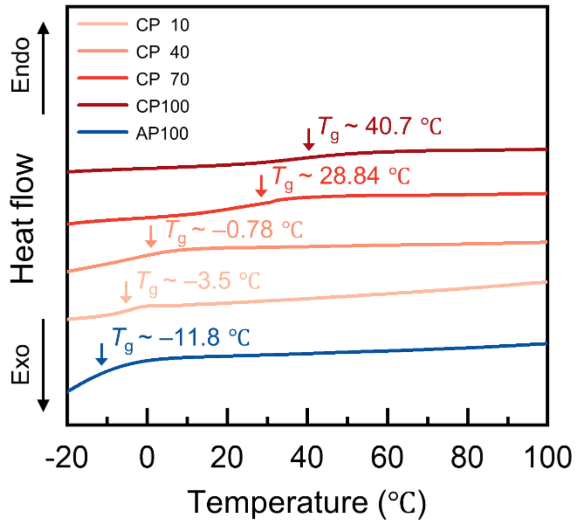

Fig. S7. Glass transition temperature (T_g) of synthesized polymers.

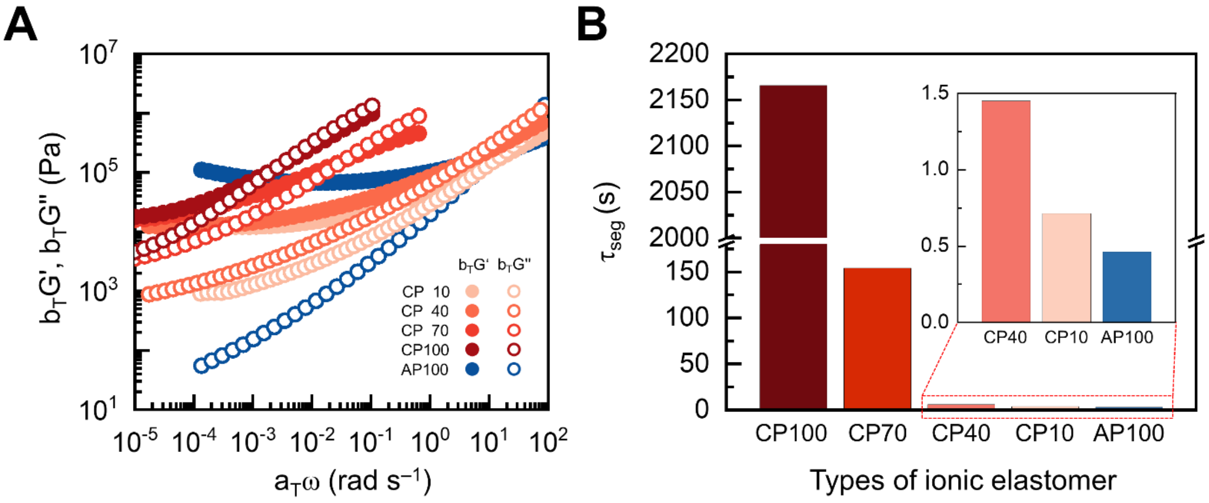

Fig. S8. **Comparison of polymeric segmental relaxation behavior according to the polymer type.** (**A**) Time-temperature superposition master based on a reference temperature of 20 °C. The inverse of the crossover frequency (τ_e) of b_TG' and b_TG'' at high frequencies define (**B**) relaxation time of entangled chain segments followed by Rouse-Zimm modes.

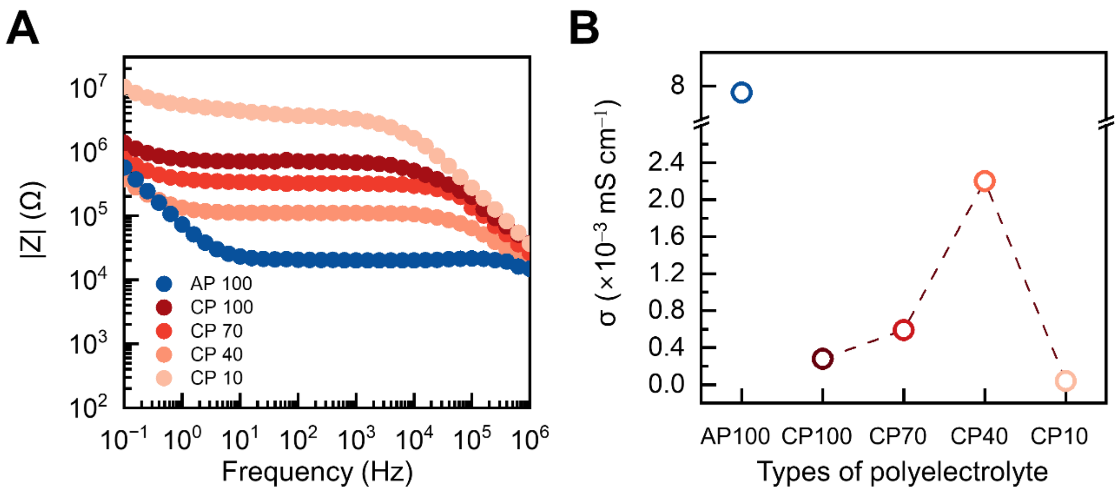

Fig. S9. **Conductivity comparison of synthesized polymers.** (**A**) Bode plots and (**B**) ionic conductivity extracted from bode plot using the equation: σ = h/AR, where h, A, and R, represent thickness, cross-sectional area, and the resistance of the polymers, respectively.

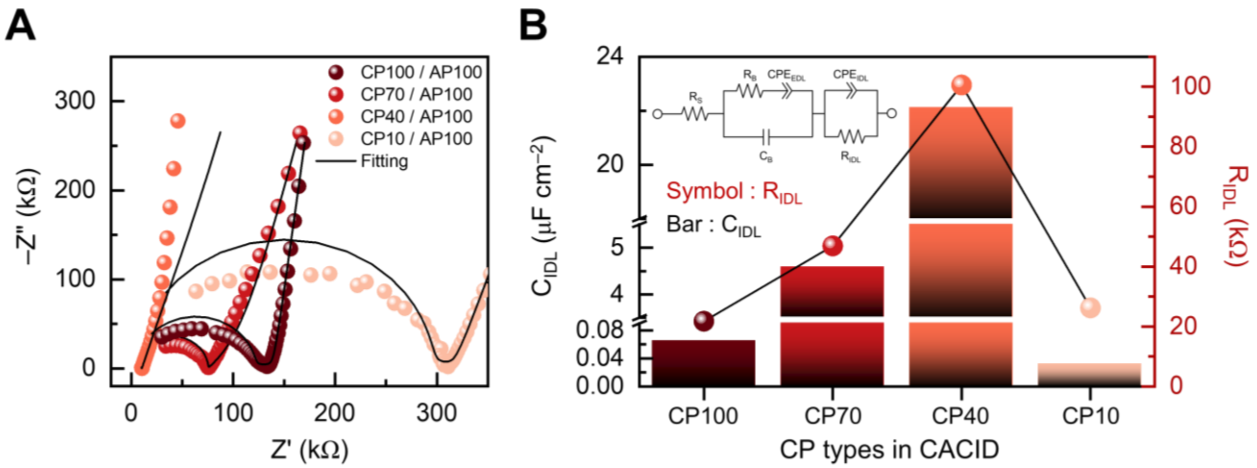

Fig. S10. **Nyquist Plot and Equivalent Circuit Analysis**. (**A**) Nyquist plot of ionic diodes according to various CPs. The semicircle in the high-frequency region represented charge transfer resistance, while the linear tail in the low-frequency region corresponded to ionic diffusion. The device incorporating CP40 exhibited a smaller semicircle and a steeper low-frequency tail, indicating enhanced ionic conductivity and reduced interfacial resistance. (**B**) Equivalent circuit model analysis of ionic diode, demonstrating that the device incorporating CP40 achieves higher capacitance. Data extracted from Nyquist plot.

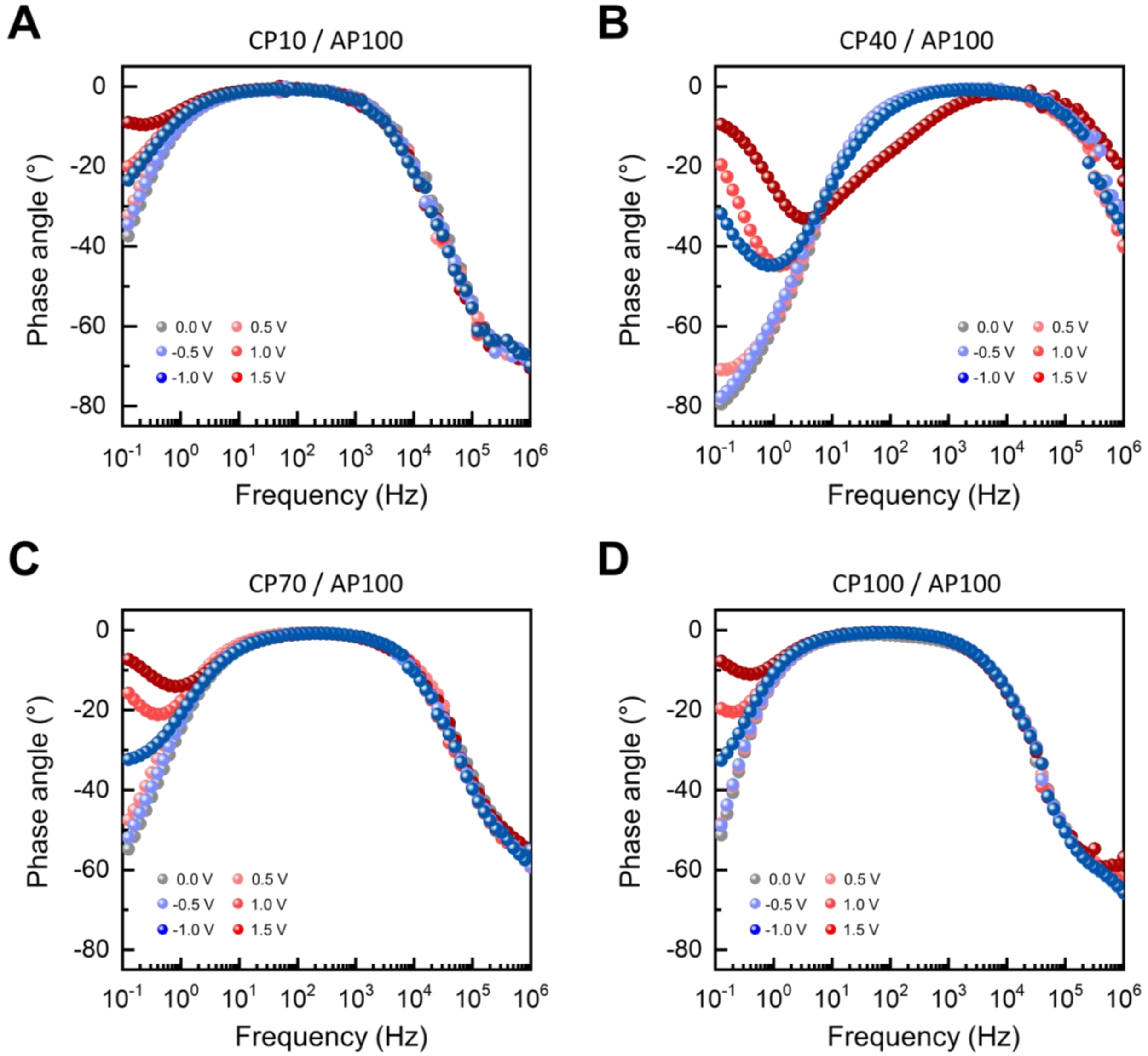

Fig. S11. **Changes in phase angle of ionic diode at various Bias.** Phase angle analysis of ionic diode based on (**A**) CP10, (**B**) CP40, (**C**) CP70, and (**D**) CP100**.**

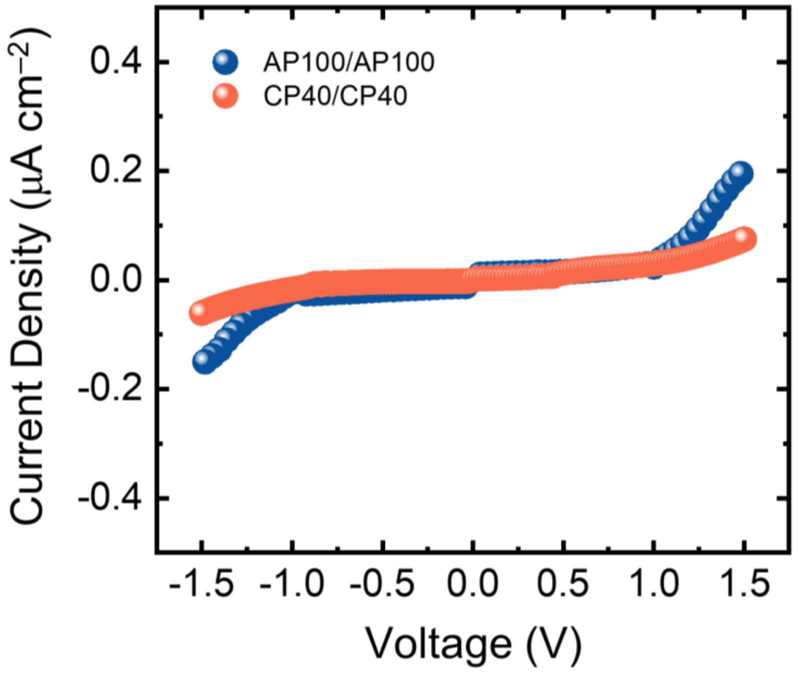

Fig. S12. I-V curves of CP40 and AP100 homojunctions.

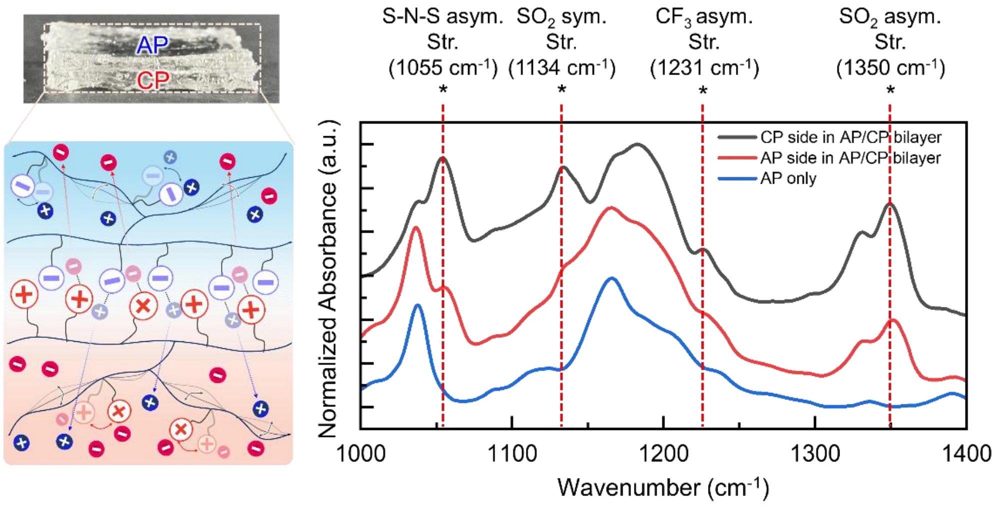

Fig. S13. ATR FT-IR analysis of counter ion diffusion across the heterojunction. ATR-FTIR spectroscopy was used to analyze the diffusion of $TFSI^-$ ions across the heterojunction. The characteristic peak of $TFSI^-$ was detected on the AP side, confirming successful formation of IDL.

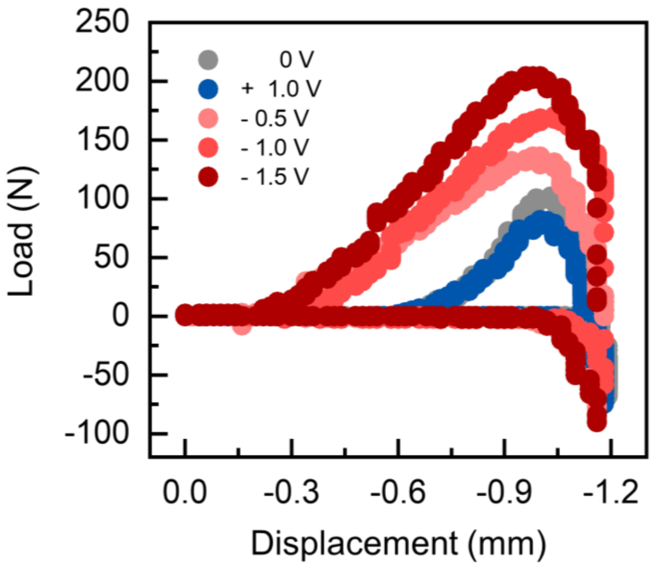

Fig. S14. Voltage-dependent adhesion behavior of CP40-based ionic diode. As the reverse bias increases, the load required to peel off the ionic diode also increases, indicating stronger interfacial adhesion. This suggests that reverse bias enhances electrostatic interactions at the interface, making delamination more difficult. In contrast, applying a forward bias of +1V does not cause any noticeable change in the load, implying that forward bias does not significantly affect interfacial adhesion.

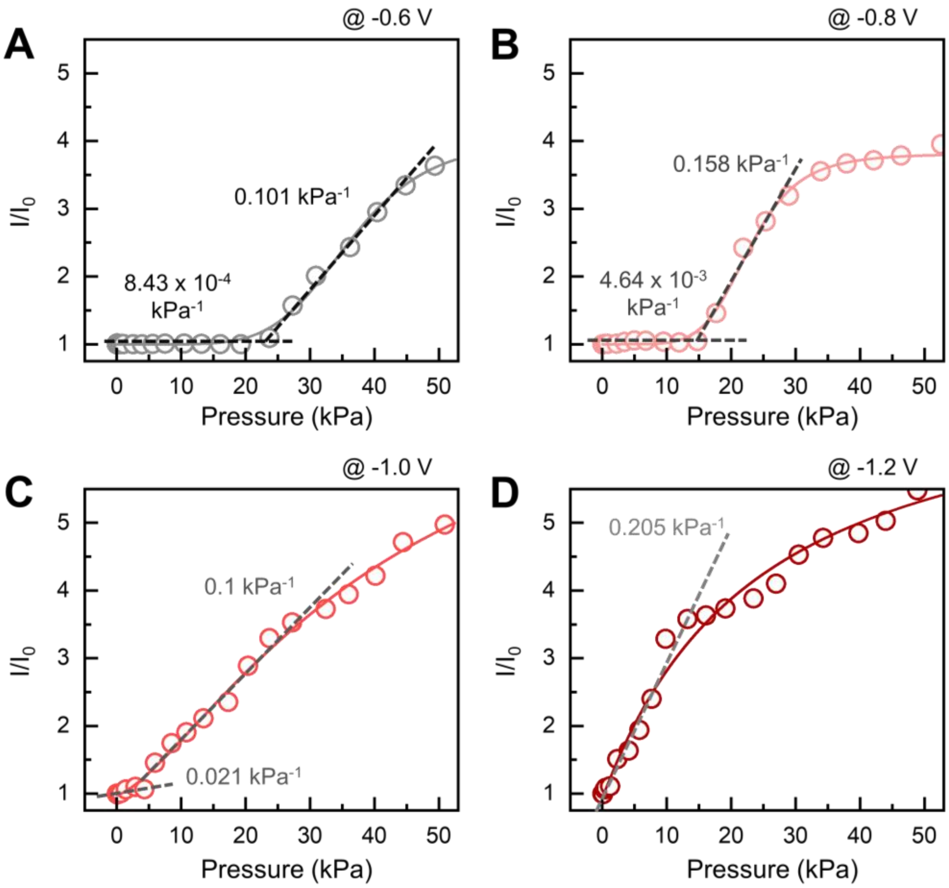

Fig. S15. Variation in pressure sensitivity reflecting threshold behavior of the CP40-based ionic diode under reverse bias conditions of (A) –0.6 V, (B) –0.8 V, (C) –1.0 V, and (D) –1.2 V.

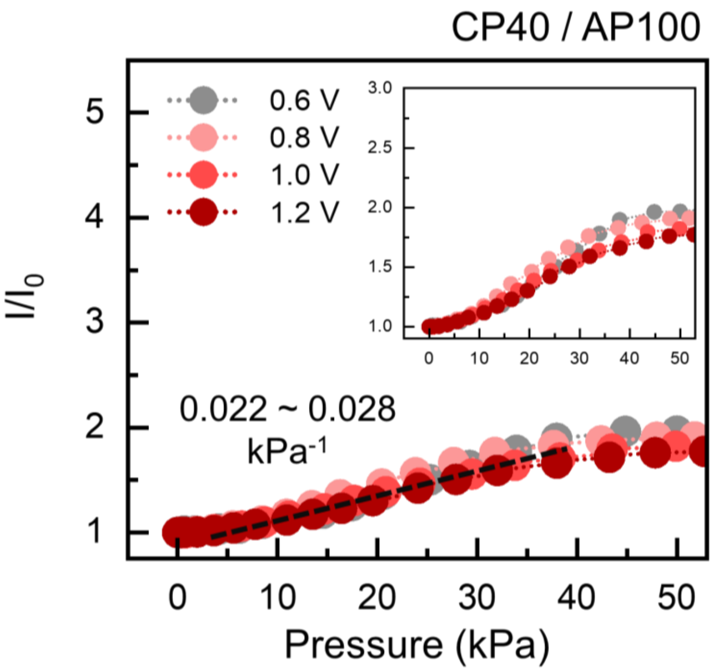

Fig. S16. Threshold behavior in heterojunction under forward bias. CP40-based ionic diode exhibits negligible current changes and sensitivity variation under forward bias.

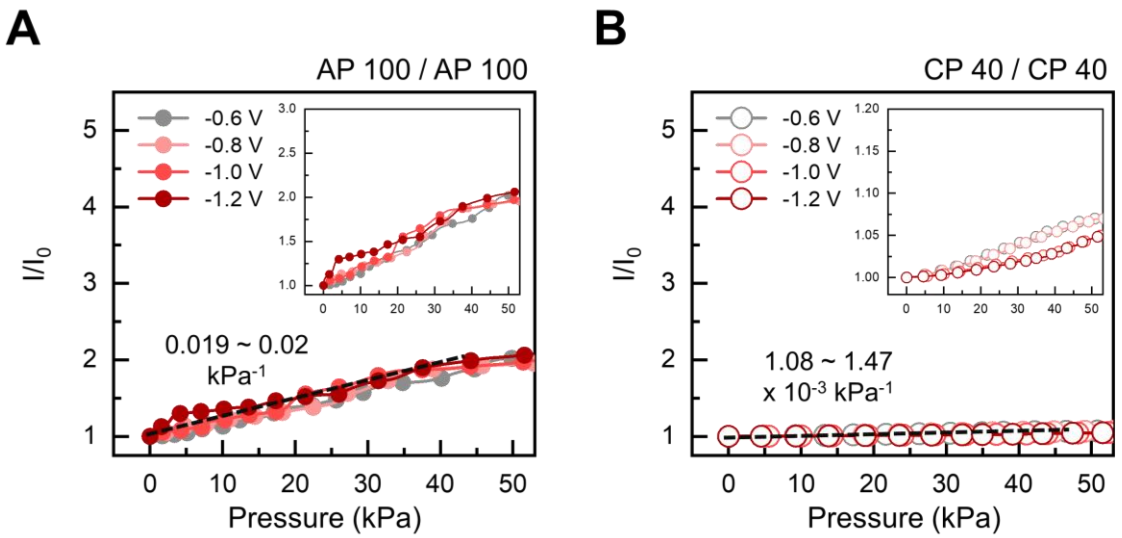

Fig. S17. Changes in current as a function of applied pressure under various reverse bias conditions. No threshold behaviors were observed in homojunction structures composed of (**A**) AP100 and (**B**) CP40.

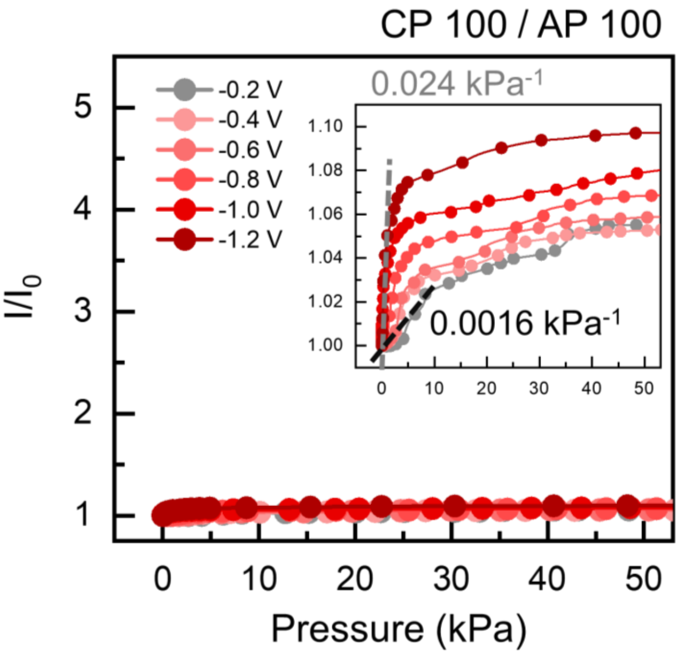

Fig. S18. Variations in current in response to applied pressure under different reverse bias conditions, demonstrating threshold behavior in heterojunction structures composed of CP100.

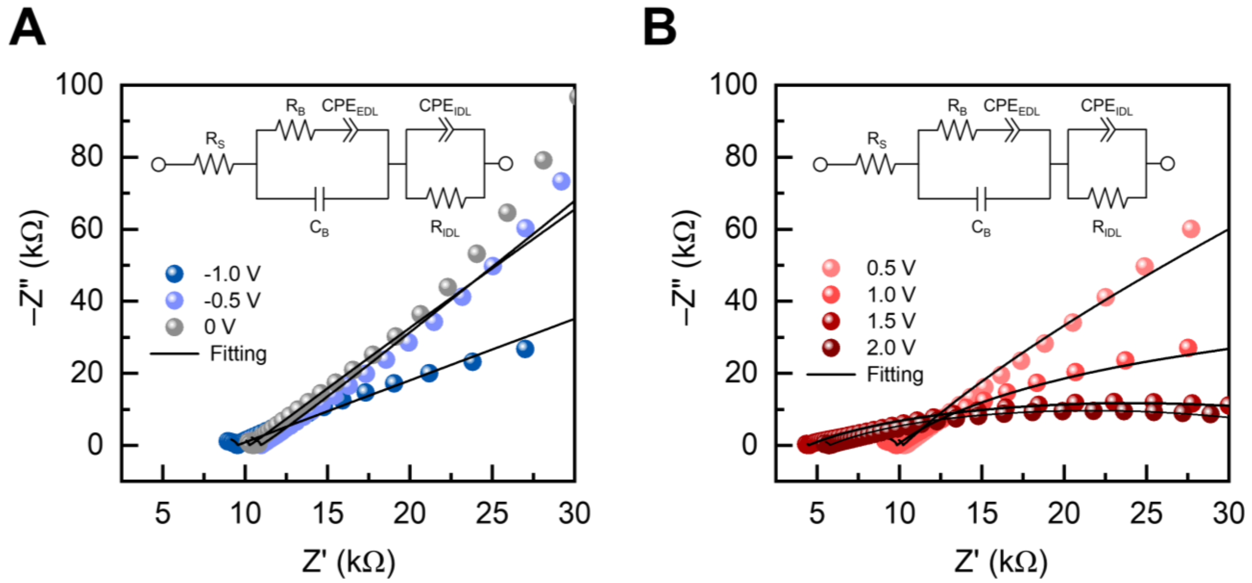

Fig. S19. Nyquist plot and equivalent circuit model analysis of ionic breakdown under various bias conditions: (**A**) Reverse bias and (**B**) forward bias on ionic diode based on CP40**.**

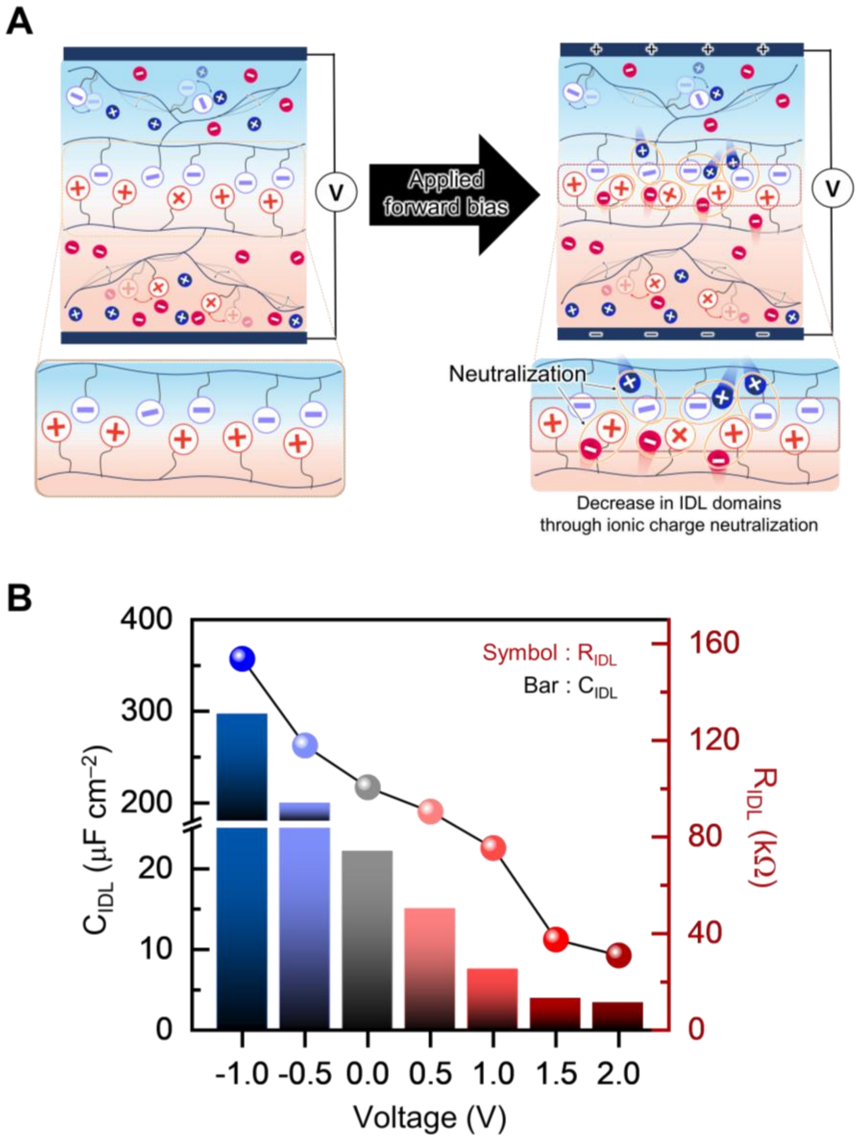

Fig. S20. Effect of forward bias on IDL capacitance and resistance. (**A**) Schematic illustration depicting the injection of mobile counter-ions into the IDL under forward bias, leading to neutralization of ionic side chains and a reduction in IDL thickness. (**B**) Measured C_{IDL} and R_{IDL} as a function of applied voltage. Under forward bias, it showed a decrease in C_{IDL}, indicating reduced charge storage capacity, and a decline in R_{IDL} due to increased ion density facilitating ionic transport.

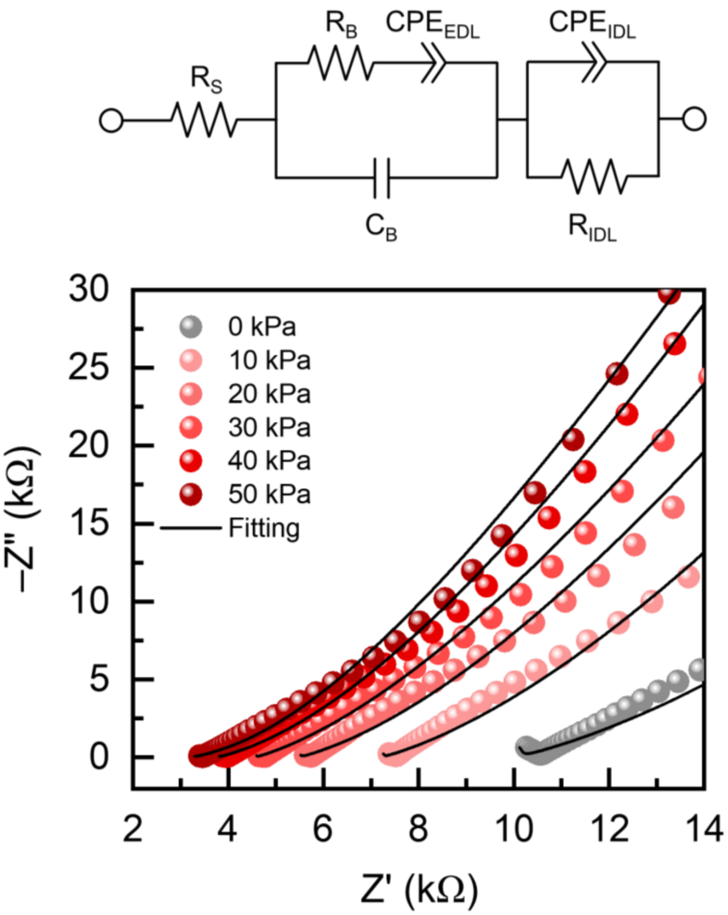

Fig. S21. Nyquist plot and equivalent circuit model analysis under various applied pressures.

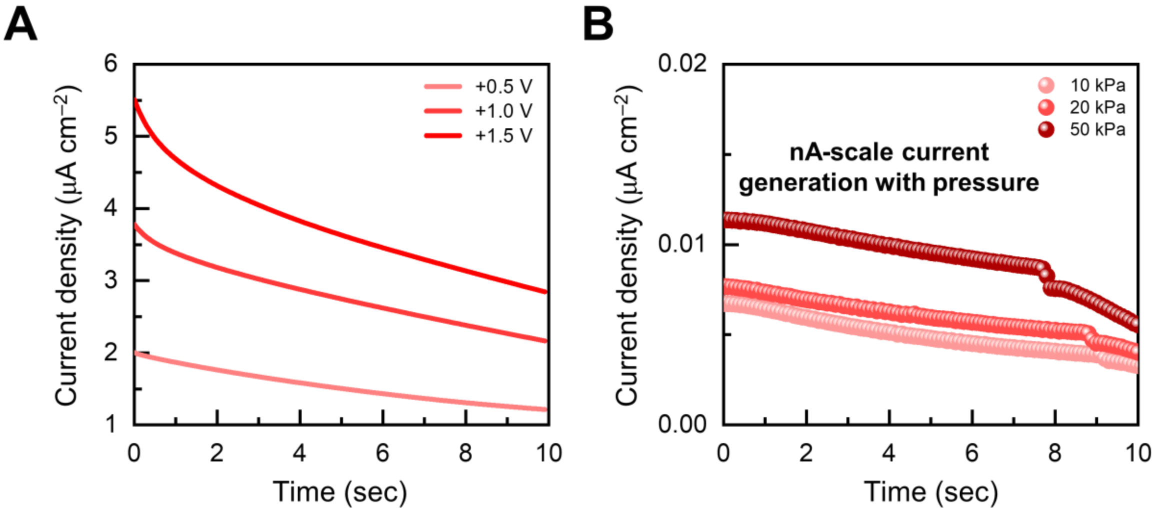

Fig. S22. Current density profiles: (**A**) Short-circuit current profiles under forward bias and (**B**) changes in open-circuit current under various pressure conditions.

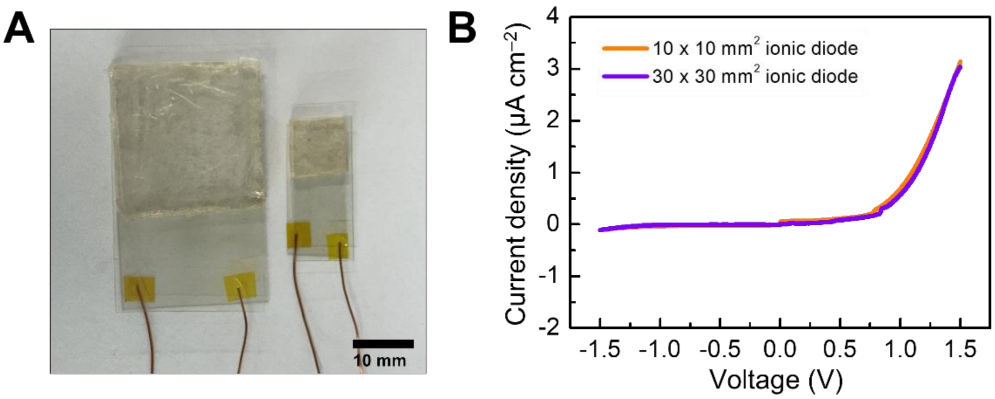

Fig. S23. Scalability of ionic diode fabrication. (**A**) Optical images of ionic diodes fabricated by monomer-solution photopolymerization with lateral dimensions of 1×1 cm^2 and 3×3 cm^2. (**B**) I-V characteristics of the corresponding devices, showing nearly identical rectification ratios, thereby confirming size-independent operation and seamless scalability.

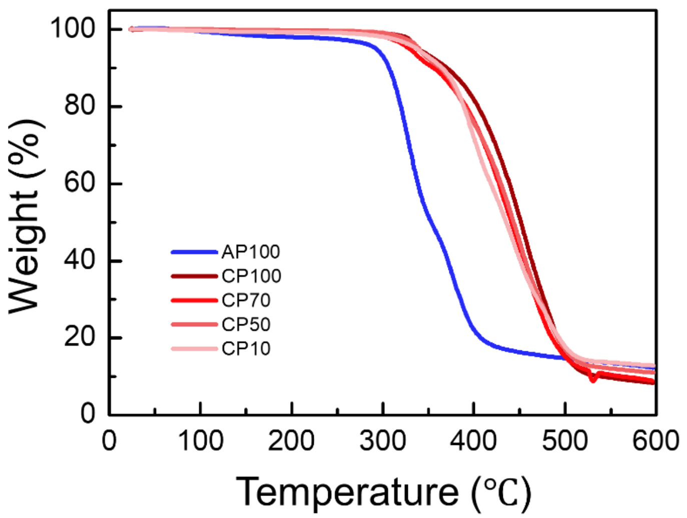

Fig. S24. **TGA curves of AP and CPs.** All samples exhibit negligible weight loss below 100 °C, confirming that no measurable moisture uptake occurs in these ionic polymers.

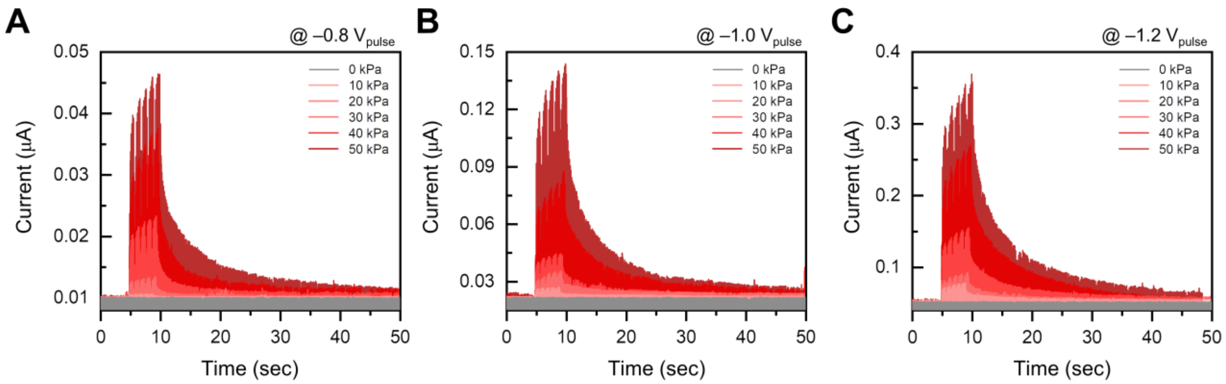

Fig. S25. Tactile perceptive current modulation in CP40-based ionic diode under pulsed reverse bias at (A) –0.8 V_{pulse}, (B) –1.0 V_{pulse}, and (C) -1.2 V_{pulse}. The current modulation was evaluated while increasing the applied pulsed pressure from 0 to 50 kPa.

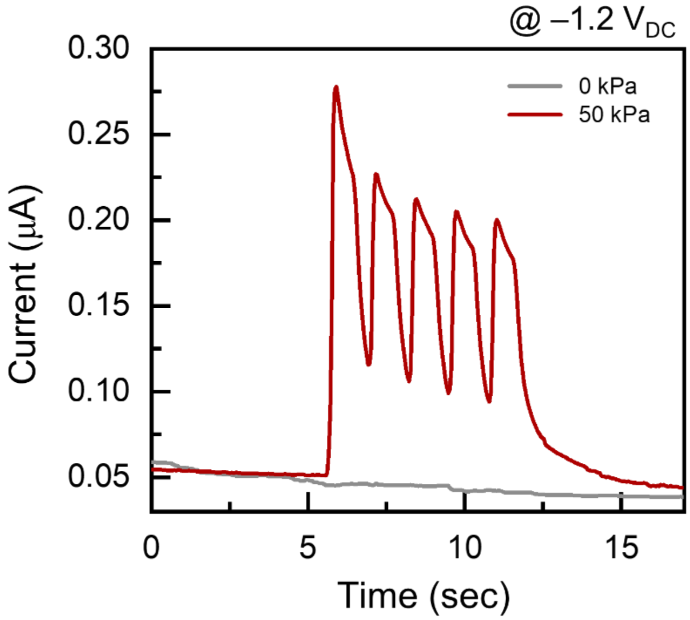

Fig. S26. Current modulation of CP40-based ionic diode under DC reverse bias (–1.2 V_{DC}) while applying pulsed pressure (0, 50 kPa).

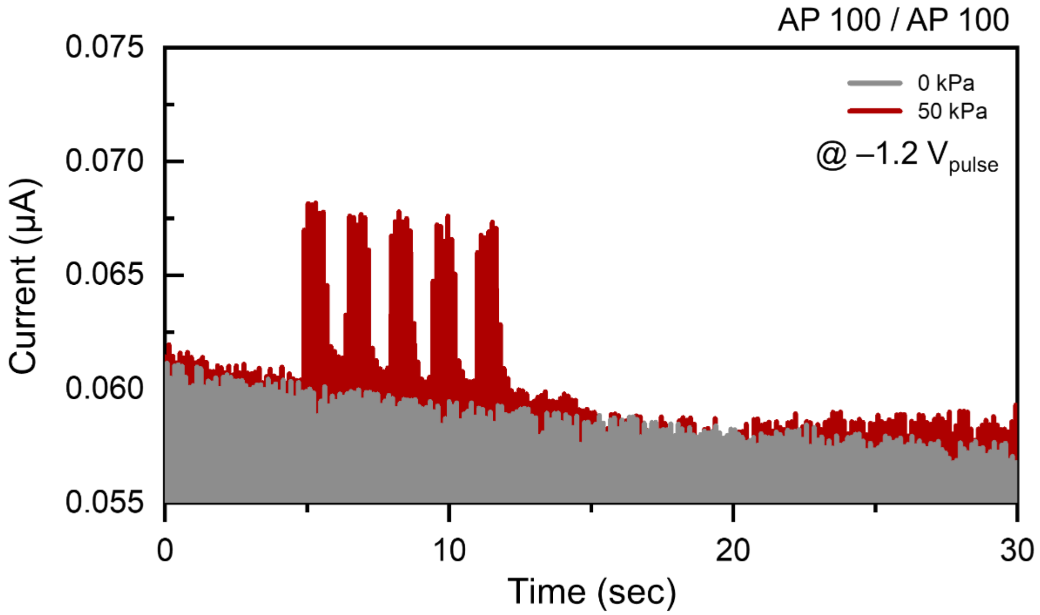

Fig. S27. Current modulation of a homojunction device composed of an APs under pulsed reverse bias (–1.2 V_{pulse}) while applying pulsed pressure (0, 50 kPa).

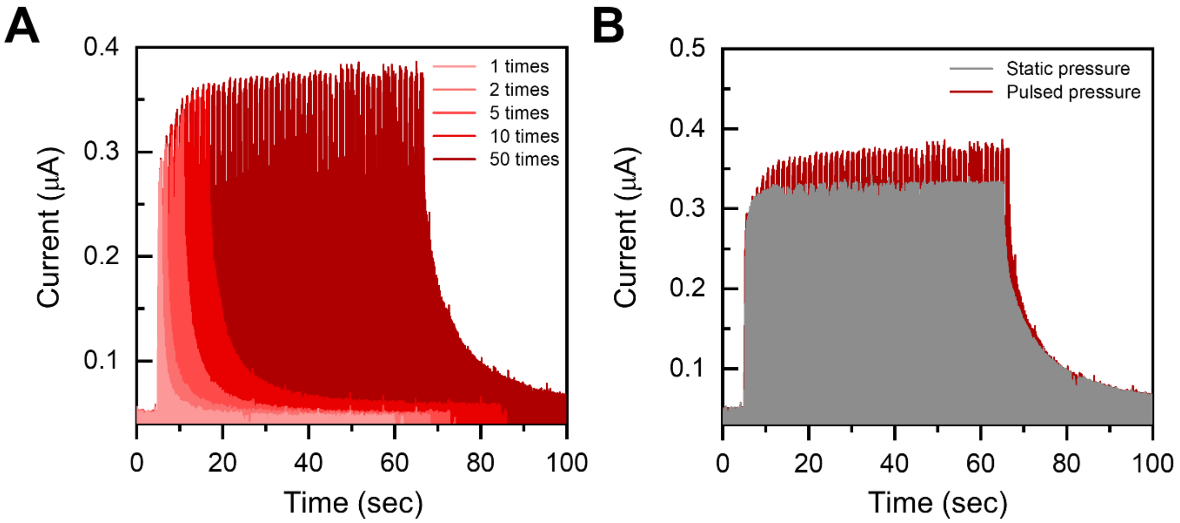

Fig. S28. Current modulation of CP40-based ionic diode under pulsed reverse bias (–1.2 V_{pulse}) while applying pulsed pressure (50 kPa) with (**A**) varying repetition numbers (1, 2, 5, 10, and 50 times). (**B**) Current modulation under pulsed reverse bias (–1.2 V_{pulse}) during pulsed or static pressure application with an amplitude of 50 kPa.

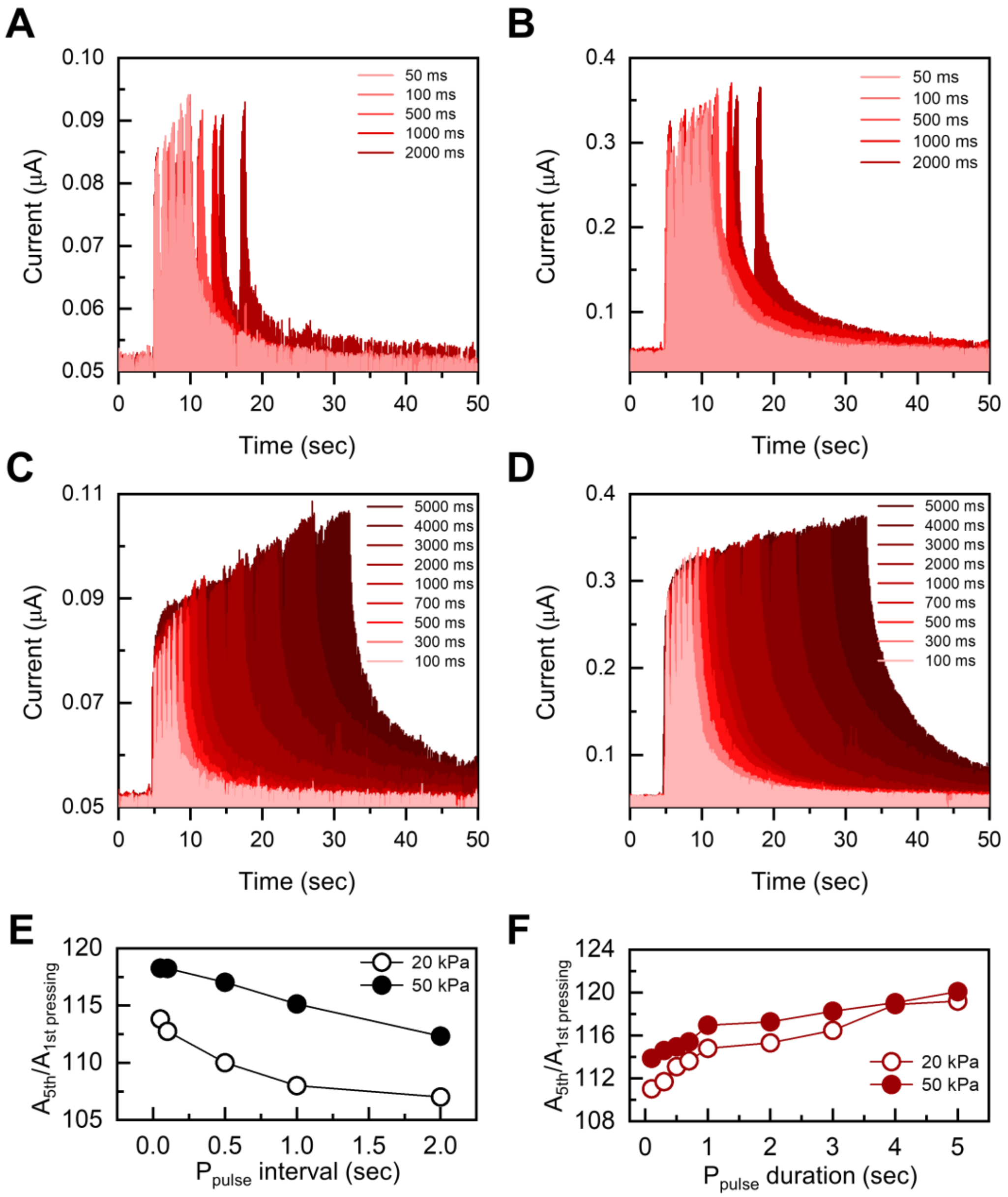

Fig. S29. Current modulation of CP40-based ionic diodes under pulsed reverse bias (–1.2 V_{pulse}) as a function of interval time and duration in applied pulsed pressure conditions. (**A, B**) Current modulation as a function of interval time for applied pressure pulses of 20 kPa and 50 kPa, respectively. (**C, D**) Current modulation as a function of duration time for applied pressure pulses of 20 kPa and 50 kPa, respectively. (**E, F**) Overall synaptic response variation with different pulse intervals and durations.

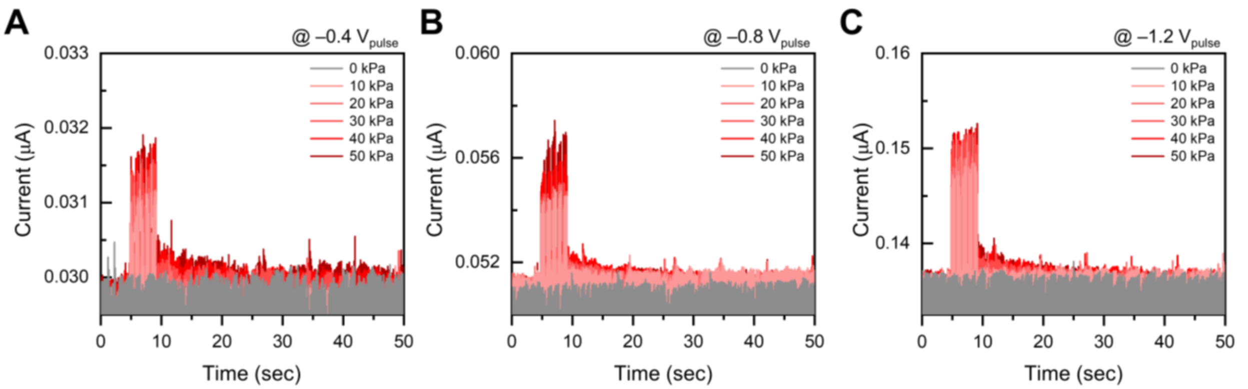

Fig. S30. Tactile perceptive current modulation in CP100-based ionic diode under pulsed reverse bias at (A) –0.4 V_{pulse}, (B) –0.8 V_{pulse}, and (C) -1.2 V_{pulse}. The current modulation was evaluated while increasing the applied pulsed pressure from 0 to 50 kPa.

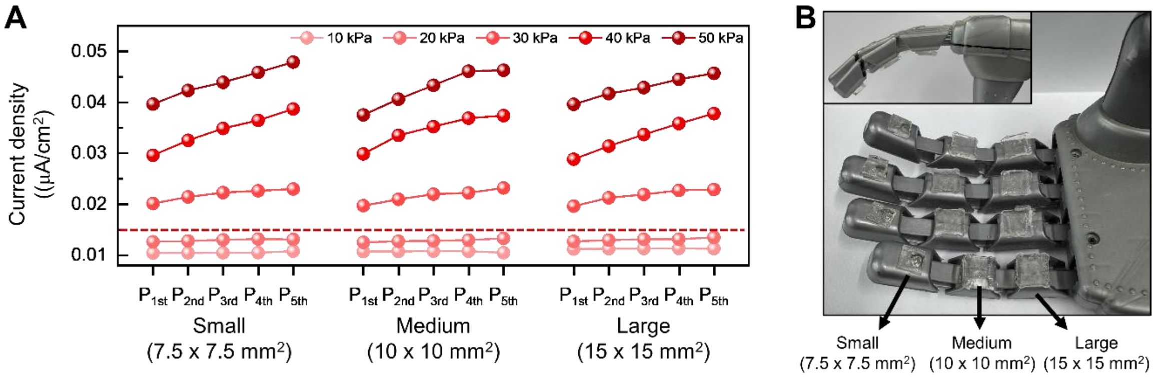

Fig. S31. Tactile perceptive current modulation in CP40-based ionic diodes with different active areas: 7.5 × 7.5 mm^2, 10 × 10 mm^2, and 15 × 15 mm^2, measured under pulsed reverse bias at –0.8 V_{pulse}. (A) The current modulation was evaluated while increasing the applied pulsed pressure from 0 to 50 kPa. (B) Photographs of CP40-based ionic diodes of varying sizes conformally attached to robotic finger segments.

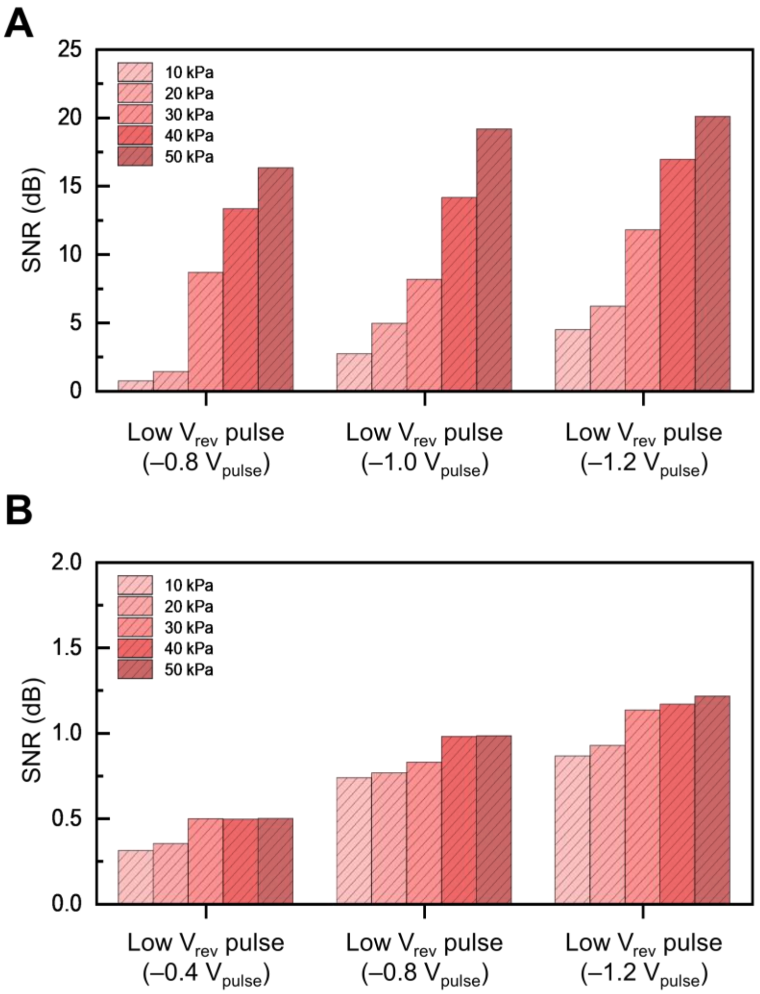

Fig. S32. Signal-to-noise ratio (SNR) characteristics of ionic diodes based on (A) CP40 and (B) CP100 under varying amplitudes of pulsed reverse bias. Each voltage value was classified into three distinct levels (low, middle, high) based on the type of cationic copolymer. The detailed calculation method was followed by the previous studies[4,45].

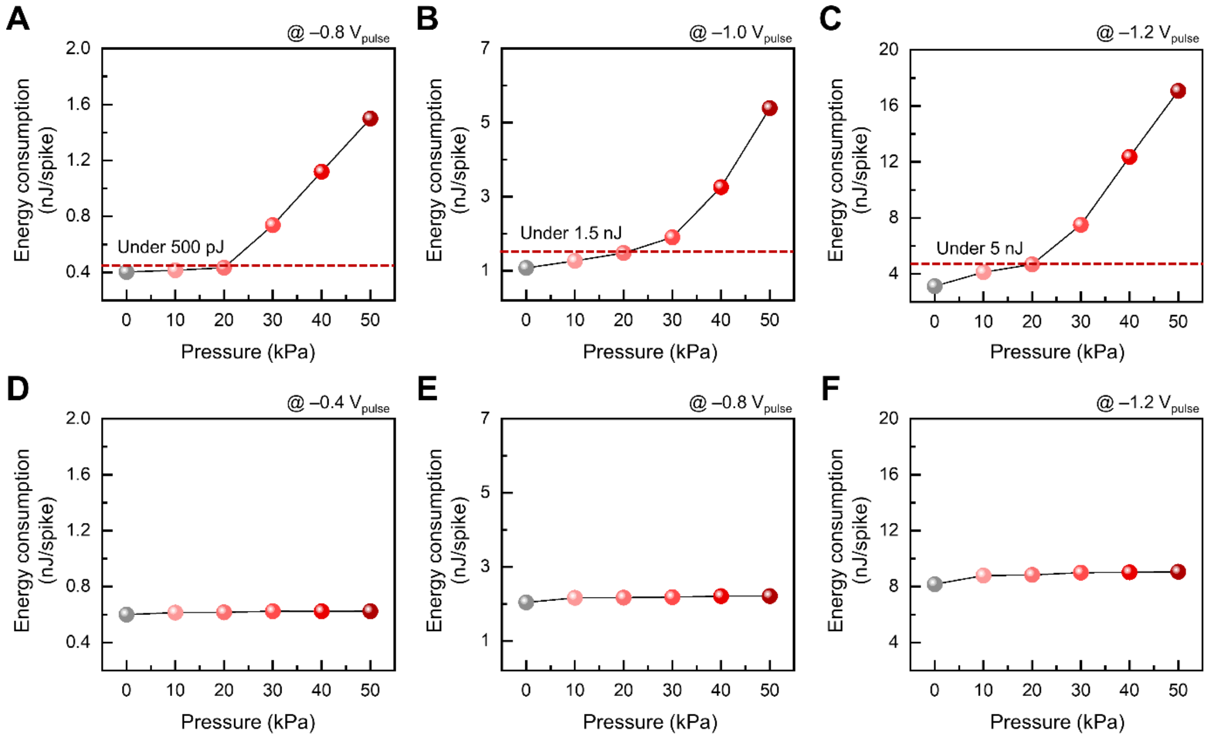

Fig. S33. Energy consumption of ionic diodes based on (A-C) CP40 and (D-F) CP100 depending on the amplitude of pulsed pressure under varying amplitudes of pulsed reverse bias. The energy consumption (E) was calculated using the following equation: $E = I_{peak} \cdot t \cdot V_{pulse}$, where I_{peak}, t, and V_{pulse} are the average value of peak current under pulsed pressure, duration time of pulsed reverse bias, and amplitude of pulsed reverse bias, respectively. The calculated energy consumption of devices was extracted from figs. S25 and S30, respectively. Each voltage value was classified into three distinct levels (low, middle, high).

Table S1.

Mass ratio of crucial tetra-components that constitute the cationic copolymer

Types of cationic polymers	Charged monomer ([AA][TFSI])	Neutral charged monomer (Butyl acrylate)	Crosslinker (PEGDA)	Photoinitiator (irgacure 2959)
CP 10	1.54 g	1.204 g	8.0 mg	1.8 mg
CP 40	2.60 g	0.74 g		
CP 70	4.10 g	0.29 g		
CP 100	5.15 g	0 g		

Table S2.

EIS fitting with CP concentration dependency

Types of ionic diode	R_S (kΩ)	R_B (kΩ)	CPE_{EDL}		C_B (pF)	CPE_{IDL}		R_{IDL}(kΩ)
			Q_{EDL}	α_{EDL}		Q_{IDL}	α_{IDL}	
CP 10 / AP 100	5.444	286.633	0.36×10^{-5}	0.803	17.6	0.12×10^{-5}	0.492	26.2
CP 40 / AP 100	1.372	8.865	0.64×10^{-5}	0.905	26.8	1.75×10^{-5}	0.708	100.6
CP 70 / AP 100	2.102	73.085	0.59×10^{-5}	0.871	30.1	0.97×10^{-5}	0.510	46.9
CP 100 / AP 100	4.487	114.587	0.60×10^{-5}	0.925	35.9	0.25×10^{-5}	0.442	21.7

*Data extracted from fig. S10 using equivalent model circuit.

Table S3.
EIS fitting with bias dependency

Bias (V)	R_S (kΩ)	R_B (kΩ)	CPE_{EDL}		C_B (pF)	CPE_{IDL}		R_{IDL}(kΩ)
			Q_{EDL}	α_{EDL}		Q_{IDL}	α_{IDL}	
–1.0	1.345	8.155	1.29 x 10^{-5}	0.663	40.9	8.79 x 10^{-5}	0.681	153.8
–0.5	1.320	9.598	0.62 x 10^{-5}	0.855	40.1	6.08 x 10^{-5}	0.623	117.8
0	1.372	8.865	0.64 x 10^{-5}	0.905	26.8	1.75 x 10^{-5}	0.708	100.6
0.5	1.372	8.826	0.90 x 10^{-5}	0.900	30.4	1.41 x 10^{-5}	0.802	90.5
1.0	1.341	8.481	15.9 x 10^{-5}	0.650	40.3	0.81 x 10^{-5}	0.846	75.4
1.5	1.350	3.106	63.9 x 10^{-5}	0.714	64.0	0.69 x 10^{-5}	0.695	37.5
2.0	1.311	4.427	76.5 x 10^{-5}	0.837	83.4	0.65 x 10^{-5}	0.700	30.9

*Data extracted from fig. S19 using equivalent model circuit.

Table S4.
EIS fitting with pressure dependency

Pressure (kPa)	R_S (kΩ)	R_B (kΩ)	CPE_{EDL}		C_B (pF)	CPE_{IDL}		R_{IDL}(kΩ)
			Q_{EDL}	α_{EDL}		Q_{IDL}	α_{IDL}	
0	1.371	8.869	0.74×10^{-5}	0.906	11.9	1.51×10^{-5}	0.651	100.5
10	1.373	5.845	0.91×10^{-5}	0.906	8.8	1.92×10^{-5}	0.653	63.1
20	1.376	3.507	1.03×10^{-5}	0.903	15.3	2.54×10^{-5}	0.642	53.0
30	1.369	3.494	1.14×10^{-5}	0.912	12.3	3.07×10^{-5}	0.663	49.5
40	1.368	2.703	1.24×10^{-5}	0.906	13.8	3.45×10^{-5}	0.627	43.8
50	1.354	2.176	1.31×10^{-5}	0.892	10.2	4.09×10^{-5}	0.577	39.6

*Data extracted from fig. S21 using equivalent model circuit.

Table S5.
Comparison of Energy-efficient performance of previously reported tactile-sensory synaptic devices

Device Type	SNR (dB)		Energy Consumption (J/spike)		Ref.
	No applied pressure (or lower applied pressure)	Applied pressure	No applied pressure (or lower applied pressure)	Higher applied pressure	
Memristor-based synaptic device (2-terminal structure)	33.66	-	2.9×10^{-9}	-	32
	34	-	21×10^{-6}	-	33
	-	-	50×10^{-9}	-	35
Transistor-based synaptic device (3-terminal structure)	-	-	0.5×10^{-9} (@ 8.71 kPa)	1.9×10^{-9} (@ 24.12 kPa)	36
	-	-	0.6×10^{-9}	-	37
	-	-	0.7×10^{-9}	8×10^{-9} (@15.68 kPa)	38
	-	-	2×10^{-9}	-	39
	15.6	-	13.2×10^{-6}	-	40
	19.45	-	5.8×10^{-6}	-	41
	7.5	34.3	0.82×10^{-9}	9×10^{-9}	4
Ionic diode (2-terminal structure)	0.69	16.32	0.41×10^{-9}	1.49×10^{-9}	This work

Supplementary Video S1 : Mechanical Threshold with synaptic data (-0.8 V vs -1.2 V in CP40-based ionic diode)

Supplementary Video S2 : Material dependency with synaptic data (CP100 vs CP40-based ionic diodes)

Supplementary Video S3 : Robotic application

REFERENCES AND NOTES

1. W. Chen, L. Zhai, S. Zhang, Z. Zhao, Y. Hu, Y. Xiang, H. Liu, Z. Xu, L. Jiang, L. Wen, Cascade-heterogated biphasic gel iontronics for electronic-to-multi-ionic signal transmission. *Science* **382**, 559–565 (2023).

2. S. Zhu, S. Chen, F. Jiang, C. Fu, T. Fu, D. Lin, Z. Meng, Y. Lin, P. S. Lee, Biopolymeric ionotronics based on biodegradable wool keratin. *Adv. Mater.* **37**, 2414191 (2025).

3. Y. M. Kim, J. H. Kwon, S. Kim, U. H. Choi, H. C. Moon, Ion-cluster-mediated ultrafast self-healable ionoconductors for reconfigurable electronics. *Nat. Commun.* **13**, 3769 (2022).

4. H. Kweon, J. S. Kim, S. Kim, H. Kang, D. J. Kim, H. Choi, D. G. Roe, Y. J. Choi, S. G. Lee, J. H. Cho, D. H. Kim, Ion trap and release dynamics enables nonintrusivetactile augmentation in monolithic sensory neuron. *Sci. Adv.* **9**, eadi3827 (2023).

5. W. Y. Choi, J. H. Kwon, Y. M. Kim, H. C. Moon, Multimodal wearable ionoskins enabling independent recognition of external stimuli without crosstalk. *Small* **19**, 2301868 (2023).

6. H. Kweon, S. Kim, B. Ha, S. Lee, S. Lee, S. Roh, H. Oh, J. Ha, M. Kang, M. S. Kang, J. H. Cho, D. H. Kim, Microlithography of hole transport layers for high-resolution organic light-emitting diodes with reduced electrical crosstalk. *Nat. Electron.* **8**, 66–74 (2025).

7. H. Na, Y.-W. Kang, C. S. Park, S. Jung, H.-Y. Kim, J.-Y. Sun, Hydrogel-based strong and fast actuators by electroosmotic turgor pressure. *Science* **376**, 301–307 (2022).

8. M. Wang, X. Xiao, S. Siddika, M. Shamsi, E. Frey, W. Qian, W. Bai, B. T. O'Connor, M. D. Dickey, Glassy gels toughened by solvent. *Nature* **631**, 313–318 (2024).

9. X. Wu, M. Ahmed, Y. Khan, M. E. Payne, J. Zhu, C. Lu, J. W. Evans, A. C. Arias, A potentiometric mechanotransduction mechanism for novel electronic skins. *Sci. Adv.* **6**, eaba1062 (2020).

10. C. Liang, C. Jiao, H. Gou, H. Luo, Y. Diao, Y. Han, F. Gan, D. Zhang, X. Wu, Facile construction of electrochemical and self-powered wearable pressure sensors based on metallic corrosion effects. *Nano Energy* **104**, 107954 (2022).

11. C. Yan, L. Xiang, Y. Xiao, X. Zhang, Z. Jiang, B. Zhang, C. Li, S. Di, F. Zhang, Lateral intercalation-assisted ionic transport towards high-performance organic electrochemical transistor. *Nat. Commun.* **15**, 10118 (2024).

12. T. Li, Z. Qu, J. Si, Y. Lee, V. K. Bandari, O. G. Schmidt, Monolithically integrated solid-state vertical organic electrochemical transistors switching between neuromorphic and logic functions. *Sci. Adv.* **11**, eadt5186 (2025).

13. F. Jiang, W. C. Poh, J. Chen, D. Gao, F. Jiang, X. Guo, J. Chen, P. S. Lee, Ion rectification based on gel polymer electrolyte ionic diode. *Nat. Commun.* **13**, 6669 (2022).

14. H. J. Kim, B. Chen, Z. Suo, R. C. Hayward, Ionoelastomer junctions between polymer networks of fixed anions and cations. *Science* **367**, 773–776 (2020).

15. S.-M. Lim, H. Yoo, M.-A. Oh, S. H. Han, H.-R. Lee, T. D. Chung, Y.-C. Joo, J.-Y. Sun, Ion-to-ion amplification through an open-junction ionic diode. *Proc. Natl. Acad. Sci. U.S.A.* **116**, 13807–13815 (2019).

16. Y. Zhang, C. K. Jeong, J. Wang, X. Chen, K. H. Choi, L. Q. Chen, W. Chen, Q. M. Zhang, Q. Wang, Hydrogel ionic diodes toward harvesting ultralow-frequency mechanical energy. *Adv. Mater.* **33**, e2103056 (2021).

17. S. W. Lee, J. Jang, Y. Kim, S. Lee, K. Lee, H. Han, H. Lee, J. W. Oh, H. Kim, T. Kim, M. D. Dickey, C. Park, Intrinsically stretchable ionoelastomer junction logic gate synchronously deformable with liquid metal. *Appl. Phys. Rev.* **9**, 041404 (2022).

18. H. Yoo, S.-B. Kang, J. Kim, W. Cho, H. Ha, S. Oh, S.-H. Jeong, S. Lee, H. Lee, C. S. Park, D.-y. Lee, T. D. Chung, K.-M. Lee, J.-Y. Sun, Ionic diode-based drug delivery system. *Adv. Mater.* **37**, e2412377 (2025).

19. O. J. Cayre, S. T. Chang, O. D. Velev, Polyelectrolyte diode: Nonlinear current response of a junction between aqueous ionic gels. *J. Am. Chem. Soc.* **129**, 10801–10806 (2007).

20. R. Peng, Y. Pan, B. Liu, Z. Li, P. Pan, S. Zhang, Z. Qin, A. R. Wheeler, X. Tang, X. Liu, Understanding carbon nanotube-based ionic diodes: Design and mechanism. *Small* **17**, e2100383 (2021).

21. M. Du, D. Zhang, W. Fan, K. Zhao, Y. Xia, Z. Nie, K. Sui, Ionic diode-based self-powered ionic skins with multiple sensory capabilities. *Mater. Today Phys.* **26**, 100744 (2022).

22. J. Yin, N. Liu, P. Jia, Z. Ren, Q. Zhang, W. Lu, Q. Yao, M. Deng, Y. Gao, MXene-enhanced environmentally stable organohydrogel ionic diode toward harvesting ultralow-frequency mechanical energy and moisture energy. *SusMat.* **3** 859–876 (2023).

23. P. He, J. H. Park, Y. Jiao, R. Ganguli, Y. Huang, A. Lee, C. H. Ahn, M. Wang, Y. Peng, Y. Long, C.-M. Chen, Z. Wang, Z. Tian, B. Mi, A. C. Arias, C. Fang, A. Toor, L. Lin, High-voltage water-scarce hydrogel electrolytes enable mechanically safe stretchable Li-ion batteries. *Sci. Adv.* **11**, eadu3711 (2025).

24. K. D. Fong, J. Self, B. D. McCloskey, K. A. Persson, Ion correlations and their impact on transport in polymer-based electrolytes. *Macromolecules* **54**, 2575–2591 (2021).

25. H. Shin, J. Shin, R. C. Hayward, H. J. Kim, Ionoelastomers at electrified interfaces: Differential electric double-layer capacitances of cross-linked polymeric ions and mobile counterions. *Macromolecules* **56**, 7827–7836 (2023).

26. H. J. Kim, L. Paquin, C. W. Barney, S. So, B. Chen, Z. Suo, A. J. Crosby, R. C. Hayward, Low-voltage reversible electroadhesion of ionoelastomer junctions. *Adv. Mater.* **32**, e2000600 (2020).

27. V. Coropceanu, J. Cornil, D. A. da Silva Filho, Y. Olivier, R. Silbey, J.-L. Brédas, Charge transport in organic semiconductors. *Chem. Rev.* **107**, 926–952 (2007).

28. J.-T. Shieh, C.-H. Liu, H.-F. Meng, S.-R. Tseng, Y.-C. Chao, S.-F. Horng, The effect of carrier mobility in organic solar cells. *J. Appl. Phys.* **107**, 084503 (2010).

29. Y. Dobashi, D. Yao, Y. Petel, T. N. Nguyen, M. S. Sarwar, Y. Thabet, C. L. W. Ng, E. S. Glitz, G. T. M. Nguyen, C. Plesse, F. Vidal, C. A. Michal, J. D. W. Madden, Piezoionic mechanoreceptors: Force-induced current generation in hydrogels. *Science* **376**, 502–507 (2022).

30. J. I. Lee, H. Choi, S. H. Kong, S. Park, D. Park, J. S. Kim, S. H. Kwon, J. Kim, S. H. Choi, S. G. Lee, D. H. Kim, M. S. Kang, Visco-poroelastic electrochemiluminescence skin with piezo-ionic effect. *Adv. Mater.* **33**, e2100321 (2021).

31. K.-H. Choi, S. J. Kim, H. Kim, H. W. Jang, H. Yi, M.-C. Park, C. Choi, H. Ju, J. A. Lim, Fibriform organic electrochemical diodes with rectifying, complementary logic and transient voltage suppression functions for wearable e-textile embedded circuits. *ACS Nano* **17**, 5821–5833 (2023).

32. Y. Xing, M. Zhou, Y. Si, C.-Y. Yang, L.-W. Feng, Q. Wu, F. Wang, X. Wang, W. Huang, Y. Cheng, R. Zhang, X. Duan, J. Liu, P. Song, H. Sun, H. Wang, J. Zhang, S. Jiang, M. Zhu, G. Wang, Integrated opposite charge grafting induced ionic-junction fiber. *Nat. Commun.* **14**, 2355 (2023).

33. R. Yuan, Q. Duan, P. J. Tiw, G. Li, Z. Xiao, Z. Jing, K. Yang, C. Liu, C. Ge, R. Huang, Y. Yang, A calibratable sensory neuron based on epitaxial VO_2 for spike-based neuromorphic multisensory system. *Nat. Commun.* **13**, 3973 (2022).

34. S. Ren, K. Wang, Y. Jia, X. Yan, Ion migration-modulated flexible MXene synapse for biomimetic multimode afferent nervous system: Material and motion cognition. *Adv. Intell. Syst.* **5**, 2300402 (2023).

35. Y. Meng, J. Zhu, Low energy consumption fiber-type memristor array with integrated sensing-memory. *Nanoscale Adv.* **4**, 1098–1104 (2022).

36. Y. Huang, Y. Zhou, S. Wieland, Y. Li, N. Zhao, J. Zaumseil, Tunable tactile synapses enabled by erasable doping in iongel-gated nanotube network transistors. *Adv. Funct. Mater.* **35**, 2423030 (2025).

37. S. Chen, Z. Zhou, K. Hou, X. Wu, Q. He, C. G. Tang, T. Li, X. Zhang, J. Jie, Z. Gao, N. Mathews, W. L. Leong, Artificial organic afferent nerves enable closed-loop tactile feedback for intelligent robot. *Nat. Commun.* **15**, 7056 (2024).

38. F. Yu, J. C. Cai, L. Q. Zhu, M. Sheikhi, Y. H. Zeng, W. Guo, Z. Y. Ren, H. Xiao, J. C. Ye, C.-H. Lin, A. B. Wong, T. Wu, Artificial tactile perceptual neuron with nociceptive and pressure decoding abilities. *ACS Appl. Mater. Interfaces* **12**, 26258–26266 (2020).

39. S. Oh, J.-I. Cho, B. H. Lee, S. Seo, J.-H. Lee, H. Choo, K. Heo, S. Y. Lee, J.-H. Park, Flexible artificial Si-In-Zn-O/ion gel synapse and its application to sensory-neuromorphic system for sign language translation. *Sci. Adv.* **7**, eabg9450 (2021).

40. X. Xie, Q. Wang, C. Zhao, Q. Sun, H. Gu, J. Li, X. Tu, B. Nie, X. Sun, Y. Liu, E. G. Lim, Z. Wen, Z. L. Wang, Neuromorphic computing-assisted triboelectric capacitive-coupled tactile sensor array for wireless mixed reality interaction. *ACS Nano* **18**, 17041–17052 (2024).

41. M. Wu, Q. Zhuang, K. Yao, J. Li, G. Zhao, J. Zhou, D. Li, R. Shi, G. Xu, Y. Li, Z. Zheng, Z. Yang, J. Yu, X. Yu, Stretchable, skin-conformable neuromorphic system for tactile sensory recognizing and encoding. *Inform* **5**, e12472 (2023).

42. J.-H. Choi, W. Xie, Y. Gu, C. D. Frisbie, T. P. Lodge, Single ion conducting, polymerized ionic liquid triblock copolymer films: High capacitance electrolyte gates for n-type transistors. *ACS Appl. Mater. Interfaces* **7**, 7294–7302 (2015).

43. G. J. Brug, A. L. G. van den Eeden, M. Sluyters-Rehbach, J. H. Sluyters, The analysis of electrode impedances complicated by the presence of a constant phase element. *J. Electroanal. Chem. Interfacial Electrochem.* **176**, 275–295 (1984).

44. M. E. Orazem, I. Frateur, B. Tribollet, V. Vivier, S. Marcelin, N. Pébère, A. L. Bunge, E. A. White, D. P. Riemer, M. Musiani, Dielectric properties of materials showing constant-phase-element (CPE) impedance response. *J. Electrochem. Soc.* **160**, C215–C225 (2013).

45. W. Xu, S.-Y. Min, H. Hwang, T.-W. Lee, Organic core-sheath nanowire artificial synapses with femtojoule energy consumption. *Sci. Adv.* **2**, e1501326 (2016).
